# Investigation of monoclonal antibody CSX-1004 for fentanyl overdose

**DOI:** 10.1038/s41467-023-43126-0

**Published:** 2023-12-05

**Authors:** Paul T. Bremer, Emily L. Burke, Andrew C. Barrett, Rajeev I. Desai

**Affiliations:** 1 Cessation Therapeutics, Inc., San Diego, CA USA; 2 Department of Psychiatry, Harvard Medical School, Boston, MA USA; 3 Behavioral Biology Program, Integrative Neurochemistry Laboratory, McLean Hospital, Belmont, MA USA

**Keywords:** Pharmacology, Addiction, Immunotherapy

## Abstract

The opioid crisis in the United States is primarily driven by the highly potent synthetic opioid fentanyl leading to >70,000 overdose deaths annually; thus, new therapies for fentanyl overdose are urgently needed. Here, we present the first clinic-ready, fully human monoclonal antibody CSX-1004 with picomolar affinity for fentanyl and related analogs. In mice CSX-1004 reverses fentanyl antinociception and the intractable respiratory depression caused by the ultrapotent opioid carfentanil. Moreover, toxicokinetic evaluation in a repeat-dose rat study and human tissue cross-reactivity study reveals a favorable pharmacokinetic profile of CSX-1004 with no safety-related issues. Using a highly translational non-human primate (NHP) model of respiratory depression, we demonstrate CSX-1004-mediated protection from repeated fentanyl challenges for 3-4 weeks. Furthermore, treatment with CSX-1004 produces up to a 15-fold potency reduction of fentanyl in NHP respiration, antinociception and operant responding assays without affecting non-fentanyl opioids like oxycodone. Taken together, our data establish the feasibility of CSX-1004 as a promising candidate medication for preventing and reversing fentanyl-induced overdose.

## Abstract (web summary)

Fentanyl continues to drive the opioid crisis by contributing to >70,000 deaths per year in the US. Here, the authors investigate a candidate medication for fentanyl overdose prevention (monoclonal antibody CSX-1004) demonstrating its mitigation of fentanyl’s effects in preclinical animal models.

## Introduction

Opioid-related overdose deaths in the United States are increasing at an unprecedented rate, continuing a two decades-long national crisis. Whereas opioid overdose deaths from 2000-2015 were due primarily to prescription opioids and heroin, the staggering uptick in opioid-related mortality is due primarily to *illicitly* manufactured fentanyl and fentanyl analogs (F/FAs). In the 12-month period ending in February 2023, 74,808 deaths were attributed to synthetic opioids (primarily F/FAs), representing 90% of all opioid-related deaths^1^. The unique pharmacology of fentanyl contributes to its exceptional overdose risk: fentanyl has high affinity and intrinsic activity at mu-opioid receptors (MOR) (>50-fold more potent than heroin) and is very lipophilic, resulting in rapid activation of central MORs to produce euphoria, respiratory depression, and other classical MOR-mediated effects^2–4^. In addition, intravenously (IV) administered fentanyl can affect respiration by causing profound rigidity in the chest wall and diaphragm as well as laryngospasm, collectively referred to as Wooden Chest Syndrome (WCS)^5–7^. The onset of WCS is extremely rapid and can complicate cardiopulmonary resuscitation attempts. As little as 2–3 mg of IV fentanyl can lead to life-threatening respiratory depression within 2 min, whereas heroin overdoses may not be lethal for at least 30 min^8^. Especially problematic is that tolerance to opioid-induced respiratory depression develops more slowly than tolerance to opioid-induced euphoria, imparting additional risks with dose escalations and more potent opioids^9^. In fact, a large portion of opioid users now prefer potent synthetic opioids over natural or semi-synthetic opioids^10^, and >60% of the illicit opioid market is dominated by fentanyl as well as ~10% by related FAs such as furanyl, fluoro, and acetyl fentanyl^11^.

The gold standard for the reversal of acute opioid overdose, including fentanyl overdose, is the opioid antagonist, naloxone (Narcan®). However, naloxone cannot be administered prophylactically, and its short duration of action demands multiple administrations to combat the effects of fentanyl and high potency FAs such as carfentanil^12,13^, although a much longer-acting naloxone-like medication known as nalmefene has been FDA-approved this year to combat F/FA overdose. Furthermore, MOR antagonists have a limited ability to ameliorate dangerous F/FA-induced WCS, as this phenomenon may not be mediated exclusively through MOR signaling^5,6^. Due to the rapid onset of fentanyl-related toxicity and the finding that many overdoses occur in isolation, the time window for administration of naloxone is often missed^8,14^. Because patients treated for opioid overdose are at high risk for a repeat overdose^15–18^, there is a pressing unmet need for novel therapeutic strategies that could provide prolonged protection from fentanyl. Such a strategy would be predicted to reduce the likelihood of overdose and bridge patients to other evidence-based treatments for opioid use disorder (OUD) (e.g., buprenorphine) that ameliorate withdrawal symptoms and craving.

In recent years, immunotherapeutic strategies have been explored to counteract the effects of synthetic opioids^19–27^. Studies have shown that endogenously produced anti-opioid antibodies from vaccination or exogenously administered anti-opioid monoclonal antibodies (mAbs) can prevent or reverse F/FA pharmacodynamics via direct sequestration of the drug in the bloodstream^19–23,27–31^. Unlike naloxone, the mechanism of action of these anti-opioid antibodies is independent of brain MOR binding due to peripheral restriction of the antibodies. Moreover, the long circulating half-life of antibodies enables their utility in opioid overdose prevention, for which there is currently no FDA-approved products, although buprenorphine has been shown to mitigate fentanyl-induced respiratory depression^32^. Herein, we profile the opioid binding activity of the first known fully human anti-fentanyl mAb (CSX-1004) and demonstrate the mAb’s efficacy for reversing and preventing fentanyl-related pharmacodynamic effects, including respiratory depression, in preclinical rodent and non-human primate (NHP) models.

## Results

### Opioid binding profile of CSX-1004

CSX-1004 is a fully human IgG1λ mAb generated using a previously reported method^23^ by immunization of OmniRat® with an opioid conjugate vaccine and sorting B-cells with opioid affinity probes. Following single-cell sequencing and replacement of rat IgC_H_ regions with human IgHG1 Fc, functional antibodies were selected based on opioid binding affinity to identify CSX-1004. Determination of CSX-1004’s binding profile was achieved by a surface plasmon resonance (SPR)-based assay in which various dilutions of fentanyl analogs were flowed across sensor-immobilized antibodies to measure k_on_ and k_off_ rates. The dissociation constant (K_D_) was then calculated as a measure of affinity and presented in Table 1 and Fig. S1 for a total of 16 fentanyl analogs. Results indicate that a wide range of FAs were recognized by CSX-1004 with picomolar affinity. A particularly large degree of flexibility was noted at the R_1_ acyl position: 1-4 carbon alkyl groups and even heteroatom-containing methoxy and furanyl groups showed picomolar affinity. Importantly, the R_1_ position is the most common area of derivatization of illicit fentanyl analogs due to the wide scope of carboxylic acids or acyl chlorides tolerated by the amide coupling reaction. The R_2_ and R_3_ positions were also readily amenable to substitutions regarding CSX-1004 binding, while R_4_ and R_5_ methylation showed a 10–100-fold reduction in affinity. Contrastingly, replacement of the R_6_ position phenyl ring caused a marked reduction (>100-fold) in binding affinity: sufentanil retained a low nanomolar K_D_ likely due to some shared chemical similarity between phenyl and thiophene rings. R_6_ substitution with the methyl ester or a tetrazolone derivative found in remifentanil and alfentanil, respectively, almost entirely abrogated antibody binding, potentially due to the disruption of van der Waals interactions between the phenethyl “tail” and the hydrophobic binding pocket when replaced with more polar groups^23,25^. Crucially, other opioids that lacked CSX-1004 cross-reactivity included morphinans such as oxycodone, opioid antagonists (naloxone, naltrexone), non-fentanyl synthetic opioids (U-47700 and methadone) as well as endogenous opioid peptides such as β-endorphin (Table 1, S1). Our in vitro binding results represent a critical predictor of CSX-1004 in vivo binding activity and opioid antagonism, which we further explored through rodent and NHP models.

**Table 1:** Opioid binding profile of CSX-1004 mAb

Fentanyl Core Structure	Opioid Name	R_1_	R_2_	R_3_	R_4_	R_5_	R_6_	k_on_ (M^−1^ s^−1^)	k_off_ (s^−1^)	K_D_ (M)	Fold- Selectivity
	Hapten	Ethyl	H	−COO-C_3_H_6_-COOH	H	H	Phenyl	ND	ND	ND	ND
	Carfentanil	Ethyl	H	−COOCH_3_	H	H	Phenyl	3.39E + 06	2.43E-04	7.18E-11	1
	Acrylfentanyl	Ethylene	H	H	H	H	Phenyl	3.43E + 06	3.06E-04	8.89E-11	1.2
	Methoxyacetylfentanyl	−CH_2_OCH_3_	H	H	H	H	Phenyl	1.37E + 07	1.58E-03	1.36E-10	1.9
	Butyrylfentanyl	Propyl	H	H	H	H	Phenyl	1.47E + 07	2.10E-03	1.43E-10	2
	p-Tolylfentanyl	Ethyl	Methyl	H	H	H	Phenyl	1.00E + 07	1.73E-03	1.73E-10	2.4
	Cyclopropylfentanyl	Cyclopropyl	H	H	H	H	Phenyl	3.61E + 06	6.57E-04	1.82E-10	2.5
	Acetylfentanyl	Methyl	H	H	H	H	Phenyl	1.10E + 07	2.13E-03	1.94E-10	2.7
	4-Fluoroisobutylfentanyl	Isopropyl	Fluoro	H	H	H	Phenyl	3.41E + 06	6.89E-04	2.02E-10	2.8
	Fentanyl	Ethyl	H	H	H	H	Phenyl	9.46E + 06	2.07E-03	2.19E-10	3.1
	4-Fluorofentanyl	Ethyl	Fluoro	H	H	H	Phenyl	3.69E + 06	1.10E-03	2.98E-10	4.2
	Furanylfentanyl		H	H	H	H	Phenyl	7.90E + 05	4.08E-04	5.17E-10	7.2
	α-Methylfentanyl	Ethyl	H	H	H	Methyl	Phenyl	6.50E + 06	4.79E-03	7.37E-10	10.3
	Sufentanil	Ethyl	H	−CH_2_OCH_3_	H	H		2.69E + 06	1.01E-02	3.77E-09	52.5
	3-Methylfentanyl	Ethyl	H	H	Methyl	H	Phenyl	1.41E + 07	9.72E-02	6.92E-09	96.4
	Remifentanil	Ethyl	H	−COOCH_3_	H	H	−COOCH_3_	ND	ND	ND	>10^3^
	Alfentanil	Ethyl	H	−CH_2_OCH_3_	H	H		ND	ND	ND	>10^4^

Fentanyl analog structures and binding kinetics as determined by SPR. The immunizing hapten used for CSX-1004 generation is shown in the first row. Fold-selectivity is calculated for each opioid relative to the carfentanil K_D_.

Cross-reactivity to non-fentanyl opioids was determined by a competitive SPR method relative to carfentanil binding. >10^4^: Naltrexone, Buprenorphine, Oxycodone, Hydrocodone, 6-Acetylmorphine. >10^5^: Naloxone, Oxymorphone, Hydromorphone, Codeine, Methadone, Morphine, U-47700, Leu-Enkephalin, β-Endorphin, Dynorphin A.

**Fig. 1:**
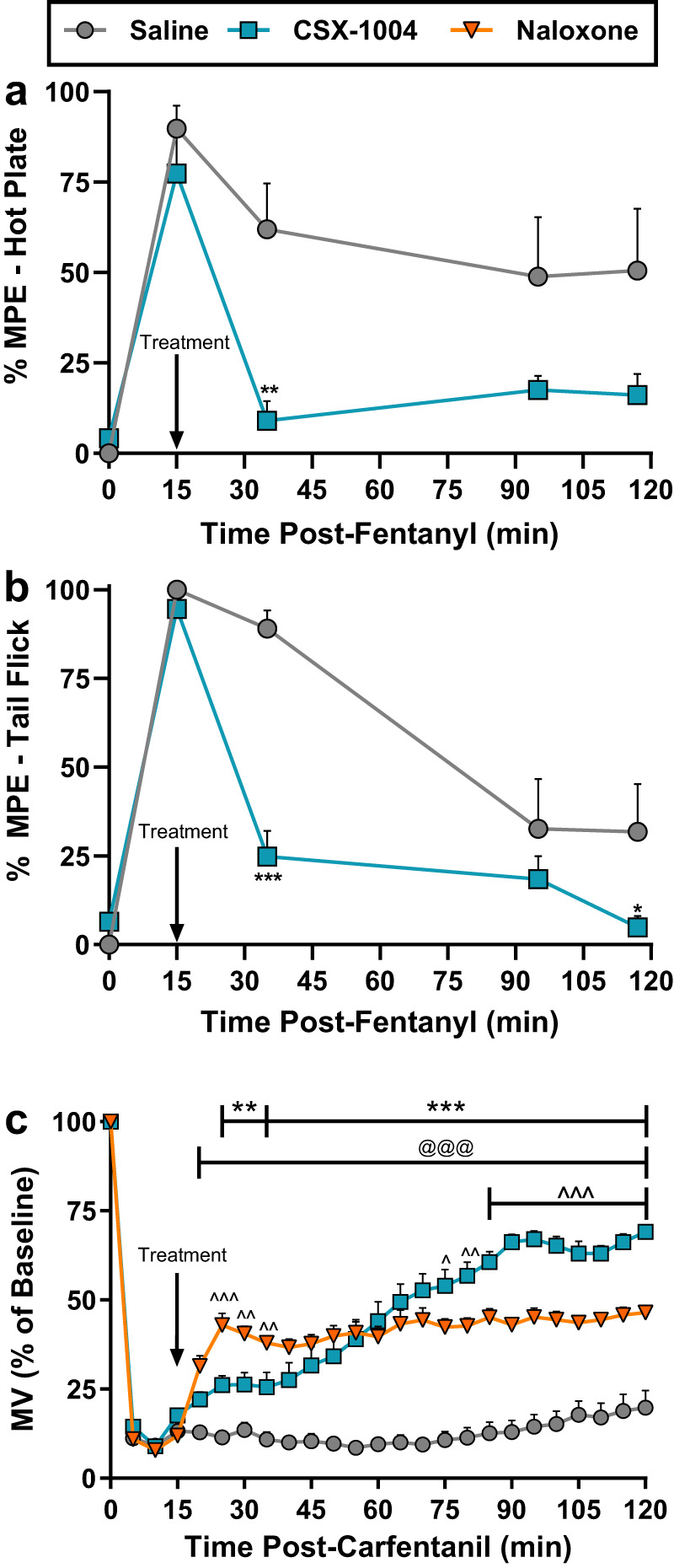
Anti-opioid efficacy of CSX-1004 in mice. Antibody-mediated reversal of fentanyl induced antinociception in (**a**) hot-plate and (**b**) tail-flick tests. Fentanyl was administered IP at 0.2 mg/kg and following a 15 min measurement, CSX-1004 was immediately administered IV at 30 mg/kg. A significant treatment effect in *n* = 6/group by two-way RM ANOVA was observed in hot plate [F (1, 9) = 7.677; *P* = 0.0217] and tail-flick [F (1, 9) = 10.98; *P* = 0.0090] assays with Bonferroni’s post-hoc test for multiple comparisons (*P = 0.0501, **P = 0.0019, ***P < 0.0001). **c** Antibody-mediated reversal of carfentanil-induced respiratory depression. Mice (*n* = 12 per group) received IV carfentanil (0.01 mg/kg) at t = 0 and IV saline, CSX-1004 (60 mg/kg), or naloxone (1 mg/kg) at t = 15 min. A two-way RM ANOVA showed a significant effect of treatment: F (2, 33) = 67.12, *P* < 0.0001 with Tukey’s post-hoc test for comparison between CSX-1004 and saline (**P < 0.01, ***P < 0.001), naloxone and saline (^@@@^*P* < 0.001), CSX-1004 and naloxone (^*P* < 0.05, ^^^^*P* < 0.01, ^^^*P* < 0.001). All data points and error bars represent means ± SEM.

### CSX-1004 mitigates fentanyl and carfentanil effects in mice

To mimic the manner in which naloxone is administered to reverse an existing opioid overdose, we evaluated the ability of CSX-1004 to reverse fentanyl-induced antinociception in mice. As shown in Fig. 1a, b, mice were administered fentanyl to induce a near 100% maximum possible effect (MPE) in hot-plate and tail-flick assays and then administered CSX-1004 via IV bolus. A marked reversal of fentanyl antinociception was observed within 20 min of mAb injection, which persisted for the remainder of the experiment. Prevention of fentanyl antinociception was also achieved when mice were pretreated with CSX-1004, and analysis of treated mouse serum indicated a strong correlation (R^2^ = 0.93) between antibody concentrations and bound fentanyl (Fig. S2). In a plethysmography procedure employed to measure opioid-induced respiratory depression (expressed as minute volume [MV]), carfentanil (an analog >10-fold more potent than fentanyl) caused a rapid, sustained drop in respiration to <20% of normal baseline values. Following treatment with IV naloxone or IV CSX-1004, respiration began to improve within 10 min compared to saline-treated animals (Fig. 1c). While naloxone was initially more effective at reversing respiratory-depression, CSX-1004 markedly surpassed the magnitude of naloxone reversal within 60 min of treatment. Reversal of fentanyl-induced respiratory depression was also achieved in this assay (Fig. S3).

### Toxicologic and toxicokinetic profile of CSX-1004

**Fig. 2:**
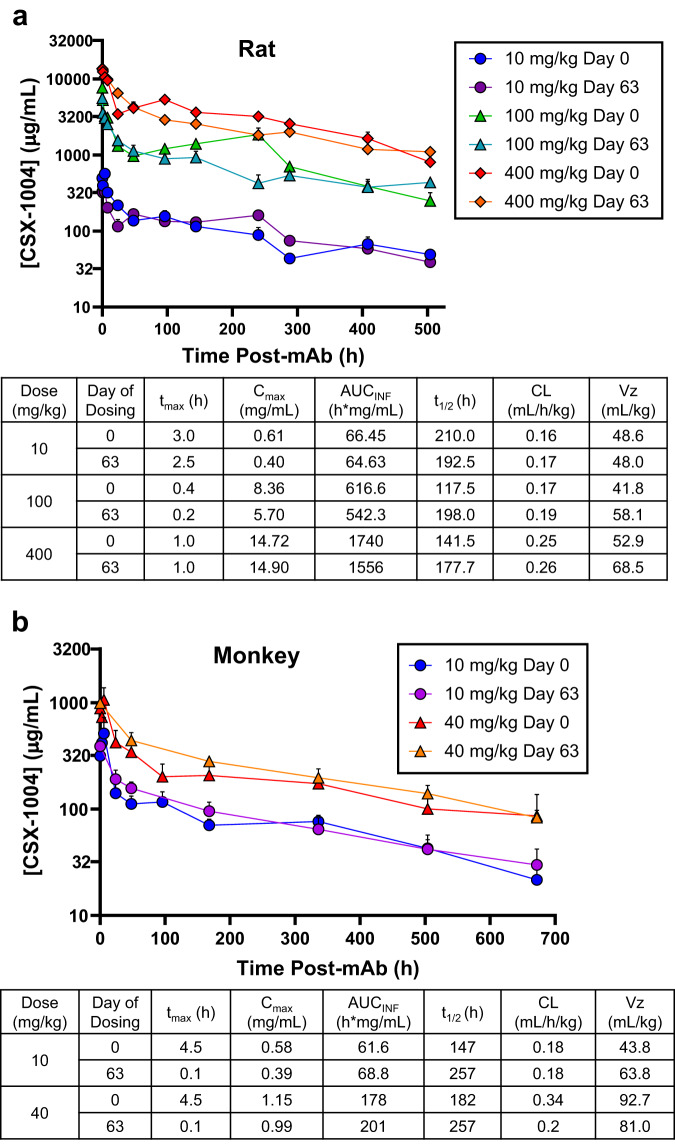
PK time courses and parameters of CSX-1004. **a** Rats (*n* = 6/sex/group) were administered CSX-1004 at the indicated doses on days 0, 21, 42, and 63 and blood sampling was performed for *n* = 3/sex/group per timepoint after the first and last administrations. **b** Antibody concentrations in monkey serum over 28-days. Two time courses were performed starting on days 0 and 63. Blood sampling was performed in *n* = 4 monkeys per time point (*n* = 3 for 40 mg/kg day 63), and the samples were analyzed by ELISA. Antibody levels on day 48 were below the limit of detection. In all cases, serum samples were measured by ELISA, and data points and error bars represent means ± SEM.

In addition to investigating efficacy, we employed a rodent model under Good Laboratory Practice (GLP) to evaluate the pharmacokinetic (PK) profile and potential toxicological effects of CSX-1004. Rats were administered 10, 100, or 400 mg/kg mAb at 0, 21, 42, and 63 days and serum concentrations of mAb were assayed after the first and last doses (Fig. 2a, Table S2). Determination of PK parameters revealed a proportional 10-fold increase in C_max_ and area under the curve (AUC) from 10 to 100 mg/kg, a 3-fold increase from 100 to 400 mg/kg, and an average elimination half-life (t_1/2β_) of 7–8 days (Fig. 2A). No significant change in PK parameters was observed from first to last dose, and importantly only 1/36 rats developed detectable levels of anti-drug antibodies (ADAs) on days 63 and 84 (Supplementary Data 1). Lastly, no adverse clinical signs or symptoms were observed, and both microscopic and macroscopic pathology workups following scheduled necropsies on days 65 and 91 showed no treatment-related effects of CSX-1004 administration establishing a no-observed-adverse-effect level (NOAEL) of 400 mg/kg (Supplementary Methods). Following the in vivo study, an in vitro tissue cross-reactivity (TCR) assay was performed to assess the potential of CSX-1004 to bind to a panel of normal human tissues, and no toxicologically significant interactions were found (Supplementary Data 1). Since monkeys were our target species for efficacy assessment, CSX-1004 concentrations in monkey serum were determined over a 28-day period following 10 and 40 mg/kg IV doses (Fig. 2b), which was repeated following a ≥ 62-day washout period. The antibody PK profile in monkeys was similar compared to the PK profile in rats albeit with a slightly longer t_1/2β_ of up to ~11 days (Fig. 2b). No significant differences were found between the day 0 and day 63 PK time courses.

### CSX-1004 provides multi-week protection from fentanyl-induced respiratory depression in NHPs

**Fig. 3:**
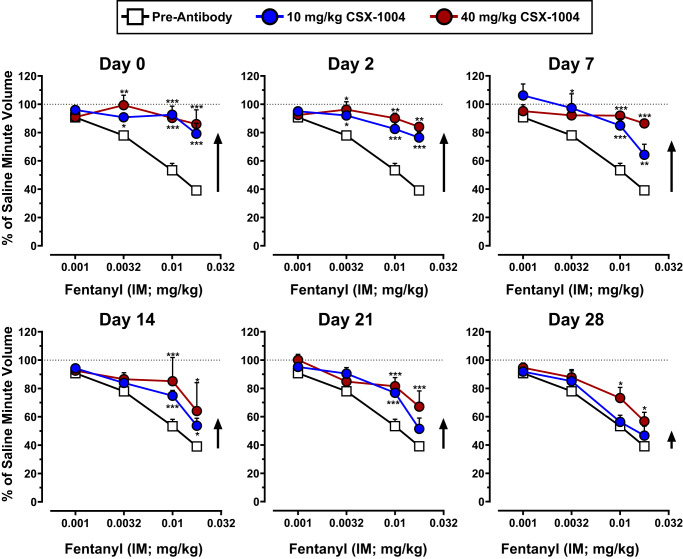
CSX-1004 provides durable protection from fentanyl respiratory depression in squirrel monkeys. Time-dependent effects of 10 mg/kg (blue circles) and 40 mg/kg (red circles) IV CSX-1004 on fentanyl respiratory depression over 28 days compared to pre-antibody baseline effects of fentanyl (white squares). Values shown are expressed as a percentage of minute volume from a saline injection (100%, dotted line) obtained on each test day from *n* = 4 monkeys (except 10 mg/kg day 7 and 40 mg/kg day 14 when *n* = 2). Arrows are shown to approximate the magnitude of CSX-1004 efficacy at each time point. Statistics were determined by a two-way RM ANOVA; 10 mg/kg effect over time [F (6, 42) = 9.348; *P* < 0.0001], 40 mg/kg effect over time [F (6, 42) = 10.22; *P* < 0.0001] with Bonferroni’s post-hoc test comparing respiration in CSX-1004-treated vs. pre-antibody at each fentanyl dose level (0.001, 0.0032, 0.01, 0.018 mg/kg) *P < 0.05, **P < 0.01, ***P < 0.001. Data points and error bars represent means ± SEM.

In addition to rodents, the ability of CSX-1004 to block the potential toxic effects of fentanyl was evaluated in a translationally relevant NHP model of opioid-induced respiratory depression (OIRD). The primate NHP model is particularly useful in that the potency of fentanyl and other opioids to depress respiration is comparable to that of humans^33,34^. Specifically, we employed a squirrel monkey (*Saimiri sciureus*) respiratory depression whole-body plethysmography procedure in which the fentanyl-dose effect curve was determined before and after IV administration of a low (10 mg/kg) and high (40 mg/kg) dose of CSX-1004 (Fig. S4). Fentanyl doses administered ranged from 0.001 to 0.018 mg/kg, the highest of which is considered potentially lethal to humans and if administered would likely require naloxone treatment^35^. Tests sessions were conducted under a normal air atmosphere as well as a mixture of 5% CO_2_ and air to increase the ventilatory response and sensitivity to opioids. Results show a significant flattening of the fentanyl dose-response curve following 40 mg/kg CSX-1004 treatment compared to pre-antibody levels, corresponding to a > 10-fold rightward shift in potency (Fig. 3). The mAb-mediated fentanyl antagonism was both dose- and time-dependent showing the greatest magnitude within the first 7 days after 40 mg/kg CSX-1004, after which the effect gradually waned through the remainder of the study. On the day 28 test session, the low-dose cohort showed a return to baseline whereas the high-dose cohort remained shifted (Fig. 3).

### CSX-1004 mitigates fentanyl potency without affecting other opioids

**Fig. 4:**
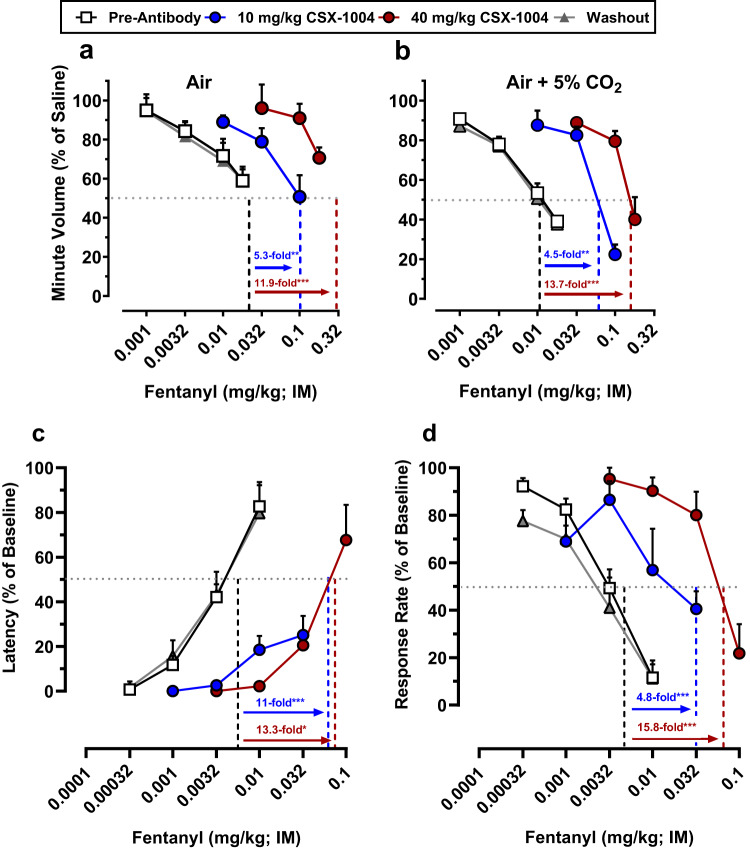
CSX-1004 shifts fentanyl dose-response curve across multiple assays. Fentanyl dose-response respiration curves under (**a**) air and (**b**) a mixture of air + 5% CO_2_ atmospheres at baseline (*n* = 8), 30 min following 10 and 40 mg/kg IV CSX-1004 (n = 4 per dose), and ≥62 days post-mAb washout (*n* = 8). Fentanyl dose-response curves in (**c**) antinociception and (**d**) operant responding procedures at baseline [(**c**) *n* = 10; (**d**) *n* = 12], 10 mg/kg and 40 mg/kg IV CSX-1004 (n = 4), and ≥62 days post-mAb washout [(**c**) *n* = 5; (**d**) *n* = 6]. Horizontal dotted lines represent 50% response and the vertical lines represent the fentanyl ED_50_ dose as calculated from each dose-response curve using linear regression analysis. The fold-shift values for each CSX-1004 ED_50_ relative to pre-antibody ED_50_ are shown above arrows including significance levels by a one-way ANOVA with Dunnett’s post-hoc test; *P < 0.05, **P < 0.01, ***P < 0.001. *P* values > 0.001 include (**a**) 10 mg/kg, *P* = 0.0069; (**b**) 10 mg/kg, *P* = 0.0018; (**c**) 40 mg/kg, *P* = 0.0310. All data points and error bars represent means ± SEM.

**Fig. 5:**
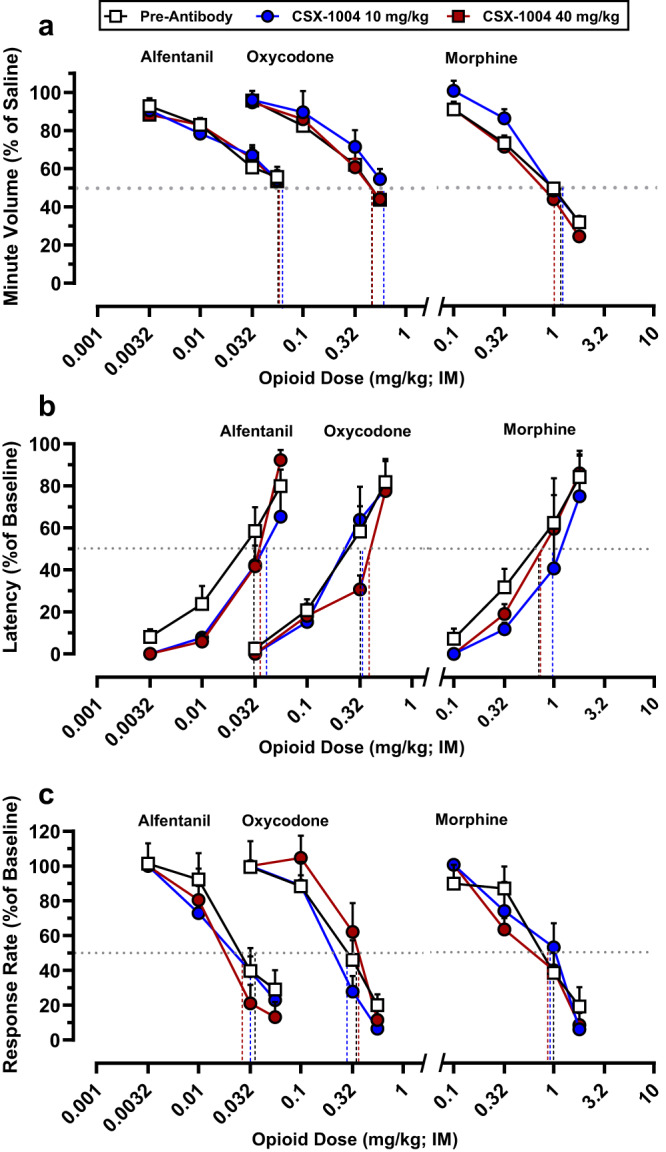
CSX-1004 does not affect pharmacology of other opioids. Opioid dose-response curves in (**a**) respiration, (**b**) antinociception, and (**c**) operant responding procedures prior to antibody administration [(**a**) *n* = 8; (**b**) *n* = 9 except *n* = 10 for oxycodone; (**c**) *n* = 12] and following 10 and 40 mg/kg IV CSX-1004 treatment (*n* = 4; except *n* = 3 for (**a**) oxycodone). Horizontal dotted lines represent 50% response and the vertical lines represent the opioid ED_50_ dose as calculated from each dose-response curve using linear regression analysis. The fold-shifts for each CSX-1004 ED_50_ relative to pre-antibody ED_50_ were not significant by a one-way ANOVA with Dunnett’s post-hoc test (*P* > 0.05). All data points and error bars represent means ± SEM.

We further investigated the therapeutic efficacy and selectivity of CSX-1004 by generating dose-response curves with high doses of fentanyl as well as non-fentanyl opioids. Pretreatment with CSX-1004 produced dose-dependent rightward shifts in the fentanyl dose-response function, resulting in >5-fold and >12-fold potency shifts vs. pre-antibody baseline for the low and high doses, respectively, regardless of plethysmograph atmosphere (Fig. 4a, b, Table S4). Remarkably, the high dose of CSX-1004 could blunt the effects of fentanyl up to 0.18 mg/kg (equivalent to 14.4 mg in an 80 kg human), doses that are likely to be lethal even in opioid-tolerant individuals who show an approximately 4-fold reduction in opioid sensitivity^9^. Following a washout period of >2 months, the fentanyl dose-response curve was redetermined and found to be equivalent to pre-antibody levels demonstrating the reversibility of mAb effects (Fig. 4a, b, Table S3). In addition to OIRD, we examined the ability of CSX-1004 to modify the antinociceptive (warm water tail withdrawal procedure) and behaviorally disruptive (food-maintained operant behavior) effects of fentanyl and other opioids^36^. Similar to the respiratory depression findings, 40 mg/kg CSX-1004 produced a ~ 13-fold rightward-shift in fentanyl’s antinociceptive and response-rate decreasing effects, which was correspondingly lower (5–11 fold) at the 10 mg/kg dose level (Fig. 4c, d, Table S3). Concurrently with the CSX-1004 treatment time course, dose-response curves in the respiratory depression, antinociception, and operant responding assays were determined for three other opioids: alfentanil, oxycodone, and morphine. Interestingly, unlike fentanyl, the pre- and post-mAb dose-response functions were essentially equivalent for all three non-fentanyl opioids demonstrating the high degree of selectivity with which CSX-1004 blocks fentanyl’s effects (Fig. 5a–c, Table S4). These observations are consistent with our predicted in vitro binding profile showing that CSX-1004 binds with picomolar affinity to fentanyl without cross-reactivity to alfentanil, oxycodone, and morphine (Table 1). Finally, CSX-1004 alone had no observable effect on normal respiration through the treatment time course (Fig. S5).

## Discussion

Antibody-based treatments for addiction and drug overdose have demonstrated positive preclinical results for over 25 years. Active vaccines have seen the most development, however nicotine and cocaine vaccines failed in clinical trials primarily due to variability of the immune response in patients^37–39^. Thus, mAbs have emerged as possibly the most promising therapeutic modality in the field of anti-drug immunobiologics because they enable control of antibody drug affinity and titer level. For example, an anti-methamphetamine mAb^40^ is being tested in phase 2 clinical trials. Fentanyl has been identified as the primary driver of deaths in the ongoing opioid crisis, and mAbs could have an enormous positive public health impact by preventing nonfatal and fatal fentanyl overdose – an unmet medical need that has not been adequately addressed with currently approved medications such as naloxone and buprenorphine.

In the present work, our in vitro binding and efficacy data in mice and monkeys support the clinical development of the CSX-1004 mAb for treatment and prevention of fentanyl overdose. Since the mechanism of action consists of antibodies binding to opioids in peripheral blood to mitigate their effects, in vitro binding assays are predictive of which opioids can be blocked by CSX-1004 in vivo. Our results demonstrate that at least 15 different FAs were recognized with high affinity. This is especially germane given that these analogs have appeared in the illicit drug supply and are predicted to be neutralized by CSX-1004. Nevertheless, fentanyl continues to make up >60% of all narcotics^11^, and 94% of all illicit synthetic opioids when also accounting for 4-fluorofentanyl^41^. Thus, targeting F/FAs with our mAb should produce the greatest clinical benefit in preventing opioid overdoses compared to targeting any other drug class. Regarding off-target binding, CSX-1004 selectively binds FAs without any cross-reactivity to endogenous opioid peptides, commonly used prescription opioids, and opioid antagonists. These data suggest compatibility of our mAb with currently available treatments for pain management, OUD, and overdose reversal. Indeed, our translational NHP experiments further confirmed this compatibility with alfentanil, oxycodone, and morphine showing no detectable alteration in their pharmacodynamic effects. While alfentanil is considered a FA, the atypical tetrazolone-containing R_6_ position “tail” instead of the typical phenethyl “tail” appeared to nullify any interaction with CSX-1004 both in vitro and in vivo; however, neither alfentanil nor any other analogs with a derivatized “tail” have been detected in the illicit drug supply. In addition, the lack of CSX-1004 interaction with alfentanil preserves the use of alfentanil for anesthesia in CSX-1004 treated patients.

A comprehensive in vivo evaluation of CSX-1004 in rodents revealed robust efficacy in reversal and blockade of opioid effects (antinociception and OIRD) that are mediated through distinct neural networks. Such findings are consistent with marked increases in peripheral concentrations of bound fentanyl in mAb-treated rodents, demonstrating a MOR-independent mechanism of action in which fentanyl is sequestered to diminish all fentanyl-mediated effects. Moreover, this mechanism was found to be effective in both prevention and reversal treatment scenarios. In the mouse respiration model, carfentanil produced a much more sustained level of OIRD than fentanyl. The dissociation half-life of carfentanil at MOR is longer relative to fentanyl (45 min versus <3 min for fentanyl), which accounts, in part, for its >10-fold higher potency than fentanyl in producing opioid-mediated effects^35^. For this reason, carfentanil induces particularly intractable respiratory depression that is not always reversible with standard naloxone doses (0.4–2 mg by injection or 4 mg intranasally)^12,13,42^. We found CSX-1004 effectively reversed carfentanil respiratory depression to a greater degree than naloxone, indicating that direct mAb-mediated redistribution and neutralization of carfentanil could be an effective strategy, either alone or in combination with naloxone, for mitigating intractable OIRD. While higher doses of naloxone above the 1 mg/kg dose used in our study may increase the magnitude of OIRD reversal, the same would be expected of CSX-1004 according to our NHP studies demonstrating dose-dependent efficacy. However, the standard naloxone dose by injection in humans of approximately 0.025 mg/kg is 40 times less than the dose we used in mice; thus, the benefit of higher naloxone doses in our particular mouse model may be limited.

In our most translationally relevant model employing NHPs, the ability of CSX-1004 to antagonize the pharmacodynamic effects of fentanyl was examined in assays of OIRD, antinociception, and operant responding. Doses of 10 and 40 mg/kg CSX-1004 produced approximately 5- and 14-fold rightward shifts, respectively, in the dose-response function for fentanyl-induced respiratory depression, and the magnitude of these shifts were similar in assays of antinociception and operant responding. These effects were selective for fentanyl, as no alterations in potency were observed for alfentanil, morphine, or oxycodone in these assays. Also, CSX-1004 proved durable in that a single IV administration of CSX-1004 provided >3 weeks protection from repeated fentanyl challenges in the OIRD assay, a finding which mirrored mAb serum concentrations in the same animals. Considering that human mAb half-life typically increases ~2-3 fold in humans from the typical ~7 day half-life in rats due to increased affinity to the human neonatal receptor (FcRn)^40,43,44^, we anticipate that CSX-1004 would be compatible with a once monthly dosing frequency. The durability of mAb-mediated antagonism of fentanyl in NHPs suggests that circulating mAb acts as a depot to temporarily sequester fentanyl away from brain MORs and regenerates free binding sites on a fast enough timescale for mitigating an additional fentanyl challenge within 48 h or sooner. Consistent with prior work, our results suggest that mAb-bound drug slowly dissociates and is metabolized via established pathways^20,45,46^, although future studies should further investigate the metabolic fate of fentanyl in the presence of CSX-1004. Moreover, normal NHP respiration in response to saline injections was unchanged throughout the study, suggesting that neither CSX-1004 nor mAb-fentanyl complexes are interacting with MORs. Compared to monkeys, humans are somewhat more sensitive to opioids (the monkey OIRD ED_50_ of ~0.02 mg/kg fentanyl is similar to the fentanyl LD_50_ in humans)^35,47,48^; thus, a larger CSX-1004-mediated fentanyl potency shift in humans could be obtained because of the higher potency of fentanyl in humans coupled with the greater predicted exposure (AUC) of the mAb in humans vs. monkeys. While models for OUD were not evaluated in this study, one could reasonably expect efficacy of CSX-1004 for OUD involving fentanyl. Indeed, clinical studies on MOR antagonist efficacy have found that a ≥ 8-fold shift in agonist potency is necessary to produce a significant treatment-related effect^49,50^.

The present findings are generally consistent with data for fentanyl conjugate vaccines, which have demonstrated positive preclinical data in mitigating opioid antinociception, overdose, and self-administration^28–31,51^, and multiple opioid vaccines are currently in development. Our initial approach to combatting fentanyl-associated risks has centered on exogenous administration of mAbs for several reasons: (a) the therapeutic effects are immediate compared to a months-long induction period with vaccines, (b) mAb affinity can be optimized and blood levels accurately titrated compared to the highly variable antibody responses from anti-drug vaccines^37–39^, (c) mAb effects are completely reversible as seen in our post-washout data, and (d) mAbs can be administered in immunocompromised patients. A potential limitation to anti-opioid mAbs vs. small-molecule medications or vaccines is the higher manufacturing and administration costs^52^, but the cost of mAbs varies greatly across therapeutic categories. Anti-opioid mAbs could reasonably be priced on par with currently marketed, once-monthly treatments for OUD. Finally, medications for OUD enjoy broad coverage by payers in the United States including Medicare and Medicaid^53,54^.

While our data provide strong evidence of CSX-1004’s utility as an overdose reversal therapy, its most useful application may be in preventing fentanyl-related overdose. We consider the 5–15 fold fentanyl potency shifts in the NHP model requiring up to 0.18 mg/kg fentanyl (equal to 14.4 mg in a 80 kg adult) to be clinically relevant for overdose prevention regardless of opioid tolerance. While chronic opioid users exhibit a 4.3-fold reduced sensitivity to fentanyl-induced respiratory depression, the doses tested in NHPs are considered lethal regardless of opioid history^9,55^. In patients who have experienced an opioid overdose or recently released from a controlled setting (e.g., in-patient detoxification program), the risk of a repeat overdose is extremely high within a 30-day period^15–18^. A single administration of CSX-1004 has the potential to provide 30-day protection during such a high-risk period. Thus CSX-1004 can provide a life-saving bridge to other treatments (e.g., buprenorphine) that address craving and withdrawal symptoms, with the option of receiving additional monthly infusions of CSX-1004. The extent, if any, that CSX-1004 may precipitate withdrawal in the setting of fentanyl dependence is currently being explored. However, the lack of competitive antagonism at MOR as well as the compatibility with withdrawal-relieving MOR agonists suggests any precipitated withdrawal symptoms could be adequately managed. Furthermore, CSX-1004, as a large molecule that does not enter the CNS, has no intrinsic abuse potential and does not impact the effects of opioids commonly employed in pain management and anesthesia.

In toxicology studies, there were no adverse effects of CSX-1004 and minimal ADA formation in rats following repeated administration of doses up to 400 mg/kg as well as no significant cross-reactivity with human tissues, providing support for initial human doses up to 40 mg/kg. However, the ADA results are limited because of the increased immunogenicity of a human mAb dosed in a rat, and the specific assay format may be biased toward detecting ADAs only against the antibody Fc portion. Thus, further assay development work must be performed for clinical testing. In addition, PK results indicate a long half-life (>7 days) in rats, and high sustained exposure over the dosing interval supports the feasibility of once-monthly dosing in humans. Coupled with the fact that >100 mAbs (many of which are the IgG1 isotype) have been FDA-approved and thus deemed to be safe, CSX-1004 shows a high degree of clinic readiness as an investigational drug and is currently being evaluated for safety and PK in a phase I first-in-human trial. If CSX-1004 is eventually approved for fentanyl overdose prevention, it could be a critical addition to the armamentarium for combatting the fentanyl crisis.

## Methods

### Study design and ethical approval

Our research complies with all ethical regulations, and all animal study protocols were approved prior to study initiation. Mouse antinociception (*n* = 6 per group) and plethysmography (*n* = 10–12 per group) studies were performed at Scripps Research Institute with 6–8 week-old female Swiss Websters (Taconic Farms, Germantown, NY) to screen the potential anti-fentanyl efficacy of CSX-1004. All mouse study protocols were approved by the Institutional Animal Care and Use Committee (IACUC) at Scripps Research Institute. The rat toxicology study performed in accordance with the principles of GLP (*n* = 40 per main study group, *n* = 12 per toxicokinetic group) was conducted with equal male and female 8 week old Sprague Dawley rats (Charles River Labs, Wilmington, MA) to assess the safety and PK of CSX-1004 at Illinois Institute of Technology Research Institute (IITRI). The rat study protocol was approved by the IACUC at IITRI. NHP whole-body plethysmography, antinociception, and operant responding assays (*n* = 4–8 per group) were performed with 5 male and 3 female 8–18 year-old squirrel monkeys (*Saimiri sciureus*, University of Texas MD Anderson Center) at McLean Hospital to assess the translational efficacy and longevity of CSX-1004 for mitigating fentanyl’s effects and its pharmacological selectivity toward other opioids. All NHP study protocols were approved by the IACUC at McLean Hospital. The GLP human tissue cross-reactivity study was approved and conducted by Charles River Laboratories in accordance with U.S. Department of Health and Human Services (HHS) and Food and Drug Administration (FDA) guidelines.

### Drugs and CSX-1004 test article

For SPR testing, all fentanyl derivatives were obtained from Cayman Chemical or Sigma Millipore as 0.1 or 1 mg/mL solutions in methanol. The peptides β-endorphin, dynorphin A (1–13), and leucine enkephalin were obtained as solids from Sigma Millipore and dissolved in deionized water at 1–10 mg/mL. Each compound solution was then diluted into the running buffer as indicated for each experiment.

For use in mouse studies, fentanyl hydrochloride, carfentanil, and naloxone hydrochloride were obtained from Cayman Chemical (Ann Arbor, MI) and dissolved into pH 7.4 phosphate-buffered saline (PBS) at a concentration of 0.02–0.04, 0.002, and 0.2 mg/mL, respectively, for administration at 5–10 mL/kg. CSX-1004 was obtained from KBI Biopharma as lot 210226-0030-M (respiratory depression) or expressed in-house via transient transfection of expiCHO cells and purified by protein A affinity chromatography (antinociception assays). Quality control analysis on in-house material was performed by SDS-PAGE to confirm purity and antibody material was concentrated to approximately 10 mg/mL by Amicon centrifugal filtration prior to mouse injection in PBS vehicle. Fentanyl-BSA coating antigen was produced in-house via chemical synthesis of a fentanyl carboxylate hapten that was conjugated to bovine serum albumin (BSA) protein via amide coupling and purified by dialysis into PBS^31^.

For use in NHP studies, fentanyl (0.001–0.18 mg/kg), alfentanil (0.0032–0.056 mg/kg), morphine (0.1–1.8 mg/kg), and oxycodone (0.032–0.56 mg/kg) were provided by NIDA/NIH Drug Supply Program (Division of Therapeutics and Medical Consequences). CSX-1004 was manufactured by KBI Biopharma (Durham, NC) on behalf of Cessation Therapeutics and provided as a ~ 100 mg/mL solution in isotonic buffer (Lot 210618-0040-BDS). Testing results of this lot met or exceeded minimum acceptance criteria for appearance (clear, slightly yellow, free of visible particles), pH, purity (SEC HPLC main peak: 98.8%, reduced CE-SDS: 97.8%), potency (100% fentanyl-BSA binding vs. reference material) and strength. Additionally, residual host cell protein, DNA, protein A as well as endotoxin were quantified and found to be below acceptable limits. Opioid solutions were freshly prepared on a weekly basis and dosed intramuscularly (IM) based on body weight. Doses of each drug are expressed in terms of the free base. CSX-1004 was thawed as needed from −80 °C storage and diluted into sterile saline for intravenous (IV) tail vein administration. Drugs were administered by IM or IV (CSX-1004) injection in volumes of ≤0.3 ml.

### Direct SPR binding assay

The binding kinetics of fentanyl derivatives were conducted on a Biacore S200 instrument equipped with a series S CM5 sensor chip. CSX-1004 mAb was immobilized on the CM5 chip surface using Amine Coupling Kit (Cytiva) as follows: (1) The active flow cell (Fc) 2 or Fc4 surface was activated for 7 min with a 1:1 mixture of 0.1 M NHS and 0.4 M EDC at a flow rate of 10 µL/min; (2) CSX-1004 was resuspended in 10 mM sodium acetate (pH 5.5) and injected over activated Fc2/Fc4 at 10 µL/min aiming at immobilization level of 1500 resonance unit (RU); and (3) The flow cell surfaces were blocked with a 7-min injection of 1.0 M ethanolamine-HCl (pH 8.5) at a flow rate of 10 µL/min. As a reference flow cell, Fc1/Fc3 was immobilized with human serum albumin (HSA, resuspended in 10 mM sodium acetate, pH 4.0) as described above aiming at the same immobilization level. The kinetics were determined via single-cycle kinetics (SCK) methodology with five analyte concentrations, and the data was fitted using a 1:1 binding model. The assay was run in PBS-P+ buffer (Cytiva) at a flow rate of 50 µL/min with a 10 Hz data collection rate as follows: (1) 3× startup cycles (each cycle includes 60 s of running buffer injection and 300 s of dissociation, all at a flow rate of 50 µL/min, the chip surface was regenerated with Gly-HCl (pH 1.5) for 30 s) were conducted before SCK analysis; (2) For binding kinetics, fentanyl, carfentanil, and related drugs were prepared in running buffer at 2.5, 5, 10, 20, and 40 nM (or 5, 10, 20, 40, and 80 nM). The compound dilutions were injected for 60 s consecutively followed by 10800 s of dissociation in running buffer; and (3) The sensor chip surface was regenerated with 30 s of injection of Gly-HCl (pH 1.5) solution before the next cycle of SCK analysis. A blank running buffer injection was also conducted before each compound run using the same conditions for SCK analysis. All data, including responses from Fc2-Fc1 or Fc4-Fc3, were collected by Biacore control software in a result file. The run data sets stored in the result file were then analyzed by Biacore S200 evaluation software (ver. 1.1 build 27) using predefined LWM kinetics/affinity single-cycle method. As shown in the appendices, Chi^2^-test values were calculated to indicate goodness of fit, SE values were calculated to indicate the significance of a parameter, and U values were calculated to indicate the uniqueness of the calculated rate constants. Each run also passed quality control as determined by the Biacore S200 evaluation software, which includes: (1) whether or not the kinetic constants are within instrument specifications; (2) whether or not the kinetics constants appear to be uniquely determined; and (3) whether or not significant bulk contribution is found.

### Competitive SPR assay

The competitive assay was run on a Biacore 3000 instrument equipped with a CM5 sensor chip. The ligand, fentanyl-BSA conjugate, was immobilized onto the chip surface using Amine Coupling Kit (Cytiva) as follows: (1) Fc2 or Fc4 surface was activated for 7 min with a 1:1 mixture of 0.1 M NHS and 0.4 M EDC at a flow rate of 10 µL/min; (2) The fentanyl-BSA conjugate resuspended in 10 mM sodium acetate (pH 4.0) were immobilized at a density of 2500 RU on Fc2 or Fc4; whereas Fc1 or Fc3 was immobilized with BSA (resuspended in 10 mM sodium acetate, pH 4.0) at a similar density to serve as reference surface; and (3) All surfaces were blocked with 7-minute injection of 1.0 M ethanolamine-HCl (pH 8.5). All assays were conducted at a flow rate of 30 µL/min at 25 °C, using 1× HBS-EP+ buffer (Cytiva) as the running buffer. CSX-1004 was pre-incubated with various compounds at room temperature for 1 h before the mixture was injected over the sensor chip surface to observe the binding. For each analysis cycle, one pre-incubated sample was injected for 300 s over all flow cells (Fc1 through Fc2, or Fc3 through Fc4), followed by 150 s of dissociation in the running buffer. The relative response at the end of dissociation (reference flow cell subtracted, Fc2 minus Fc1 or Fc4 minus Fc3) was recorded in the sensorgram and was used for binding evaluation. After each sample analysis, all flow cells were regenerated with 30 s injection of 10 mM Gly-HCl, pH 1.5, before next cycle of analysis. Triplicate measurements were taken for each competitor compound.

### Mouse study subjects

Female Swiss Webster mice (6–8 weeks old, Taconic Farms) were housed in a climate-controlled vivarium [(temperature = 72 °F, range: ~64–84 °F); relative humidity = 50%, range: 40–70%] on a 12 h light/dark cycle (lights on at 7 AM–7 PM) with ad libitum access to water and food (Inotiv #2018). Euthanasia was performed post-study via carbon dioxide asphyxiation followed by cervical dislocation in accordance with American Veterinary Medical Association (AVMA) guidelines.

### Reversal of fentanyl antinociception in mice

CSX-1004 was tested for its ability to reverse the effects of fentanyl in both the hot-plate and tail-flick antinociception tests. First, fentanyl (0.2 mg/kg, IP) was administered at Time 0 to *n* = 6 mice/group, and CSX-1004 (30 mg/kg, slow bolus IV) or saline was administered 15 min later. Antinociceptive testing was performed at 0, 15, 35, 95, and 115 min post-fentanyl administration.

For all experiments, baseline nociceptive responses for each mouse were measured immediately prior to starting each experiment in the hot-plate and tail-flick assays, followed by testing at the aforementioned timepoints with the hot-plate conducted first immediately followed by tail-flick. For the hot plate assay, the mouse was observed in an acrylic cylinder (14 cm diameter × 22 cm) on a 55 °C hot plate surface. The latency to perform one of the following nociceptive responses was measured: licking of the hind paw, shaking/withdrawal of the hind paw, or jumping. A 35-second cutoff was used to prevent tissue damage.

For the tail flick assay, the latency to tail withdrawal from a heated light beam (45% active intensity) (IITC Life Science Tail Flick Analgesia Meter, Woodland Hills, CA) was measured. A 10-second cutoff was used to prevent tissue damage.

Percent maximum possible effect (%MPE) was calculated for each post-baseline time point by the following equation: $\%{MPE}=\frac{({test}-{baseline})}{({cutoff}-{baseline})}\times 100$

### Reversal of respiratory depression in mice

CSX-1004 was tested for its ability to reverse the effects of carfentanil and fentanyl using a plethysmograph apparatus from EMKA Technologies (Paris, France) under a 5% CO_2_ in air atmosphere equipped with whole-body chambers designed for mice. Mice were given a 20 min equilibration period in the plethysmograph chambers while observing respiration for baseline measurements. Next, they were quickly removed to administer an IV dose of carfentanil (0.01 mg/kg, *n* = 12/group) or fentanyl (0.135 mg/kg, *n* = 10/group) given retroorbitally at *t* = 0 min and placed back in the chambers. Respiration was observed to decrease for a 15 min period after which the mice were again removed and administered IV saline, IV 60 mg/kg CSX-1004, or IV 1 mg/kg naloxone retroobitally on the opposite side that the opioid was given. After placing the mice back in the chambers, respiration was observed for an additional 105–120 min. Using the IOX2 software by EMKA, data were collected continuously and binned in 5-min intervals for data management and analysis purposes. Minute volume (MV), tidal volume (TV), and respiration rate (f) data were collected and are related by the equation: MV = TV X f. For each mouse, minute volume data from the 20 min equilibration period was averaged and designated as the 100% of baseline value while subsequent data points were normalized within subject to this value by the equation: MV/MV_baseline_ X 100 = MV_% of baseline_.

### NHP study subjects

A group of eight adult squirrel monkeys (5 Males/3 Females; *Saimiri sciureus*, 8–18 years, 650–1000 g, University of Texas, MD Anderson Center) were housed in a climate-controlled vivarium [temperature = 72 °F, range: ~64–84 °F; relative humidity = 50%, range: 30–70%] with a 12-h light/dark cycle (lights on at 07:00 AM–7:00 PM) in the McLean Hospital Animal Care Facility. The facility is licensed by the United States Department of Agriculture of the Institute of Laboratory Animals Resources, Commission on Life Sciences (National Research Council, 2011). Except during experimental sessions, subjects had ad libitum access to water. Food intake was not restricted in this study; however, after daily weighing, diets were adjusted as needed for each subject to maintain a constant level of body weight. All subjects were fed a high-protein primate chow (Purina Monkey Chow, St. Louis, MO) supplemented with fruit and multivitamins. All experiments were conducted five days a week (Monday to Friday) between 8:00 AM and 2:00 PM in accordance with guidelines established by the National Institutes of Health Committee on Laboratory Animal Resources. Following completion of the studies, NHP subjects were returned to the animal colony at McLean Hospital for reassignment to other research projects.

### NHP respiration study apparatus

Respiration studies were conducted as described previously^56^ in a custom-made acrylic chamber (9.5”d × 10”w × 10”h) that served as a whole-body plethysmograph (EMKA Technologies, Montreal, PQ, Canada). A continuous 5 L/min flow of balanced air or air mixed with 5% CO_2_ was introduced through a port at the front of the chamber and removed at the same rate through a similar port in the rear of the chamber. Changes in flow inside the plethysmograph were recorded by a pressure transducer connected to a computer; SCIREQ IOX2 software converted pressure displacement to measures of breathing frequency, tidal volume, and minute volume.

### NHP respiration procedure

This respiration study was conducted in five phases which included the determination of (see Fig. S4): (1) the baseline respiratory response induced by fentanyl and other opioids; (2) the mitigation of the fentanyl respiratory response by pre-treatment with CSX-1004; (3) the re-establishment of baseline fentanyl respiratory response following a ≥ 62-day washout period post-CSX-1004 administration; (4) the mitigation of the opioid respiratory depression with higher doses of fentanyl by a re-administration of CSX-1004; and (5) the selectivity of CSX-1004 by evaluating its ability to block the respiratory depressant effects produced by three other opioids.

To generate opioid dose-response functions in the respiratory depression assay, experimental sessions consisted of 5 successive test cycles. An IM saline injection was given immediately prior to the first cycle, which included respiration measurements in air (20 min) followed by 5% CO_2_ in air (10 min). The extended period within the air condition during the first cycle was to allow for habituation to the chamber and conditions. Next, up to four IM cumulative doses of the opioids given were followed by measurement in the plethysmography chamber in air (10 min) then a mixture of 5% CO_2_ and air (10 min). Drug injections were administered following each exposure to the 5% CO2 and air mixture, and respiratory rate and tidal volume (ml/breath) were recorded over 1 min periods and multiplied to obtain minute volumes. The range of fentanyl doses was adjusted based on the antibody treatment schedule to account for rightward shifting of the dose-response function but was always preceded by a saline injection and respiratory measurements. The effects of saline and a wide range of doses of fentanyl (0.001–0.018 mg/kg), alfentanil (0.0032–0.056 mg/kg), oxycodone (0.032–0.56 mg/kg), and morphine (0.1–1.8 mg/kg) on respiration were determined prior to CSX-1004 administration.

Once baseline measurements were established, CSX-1004 was intravenously (IV) administered to squirrel monkeys via slow bolus into the tail vein on Day 0 at low (10 mg/kg, *n* = 4; 2/sex) and high (40 mg/kg, *n* = 4; 3 male, 1 female) dose levels. The animals were then re-tested using the above-described respiration procedure with one saline injection followed by four injections of increasing doses of fentanyl (0.001 to 0.018 mg/kg). The identical fentanyl challenge testing procedure occurred six times over a 28-day period starting at 30 min (Day 0) post-mAb dose as well as Days 2, 7, 14, 21, and 28 to determine the duration of CSX-1004’s action. To account for small variations in normal respiration over time, minute volumes were normalized as a percent to the within-subject saline response minute volume gathered on each given test day.

On each of the fentanyl dosing days from the first experiment (see above), whole blood samples were collected, processed to serum, and analyzed for CSX-1004 concentrations (see method description below). Additional blood sampling was performed on the first day of CSX-1004 dosing at 5 min, 3 h, 6 h and 24 h, and on Day 48; however, the Day 48 samples showed no detectable levels of antibody.

Following a 62–128-day washout period and re-establishment of the fentanyl alone dose-response curve, monkeys were administered a second IV CSX-1004 dose of either 40 mg/kg (*n* = 4; 3 male, 1 female) or 10 mg/kg (*n* = 4; 2/sex) and the fentanyl dose-response curve was determined at fentanyl doses of 0.032 to 0.18 mg/kg or 0.01 to 0.1 mg/kg, respectively.

In the next phase of the study, the selectivity of CSX-1004 was evaluated by assessing its impact on the three opioids with potent respiratory depressant activity (alfentanil [40 mg/kg: *n* = 4; 3 male, 1 female, 10 mg/kg: *n* = 4; 2/sex], morphine [40 mg/kg: *n* = 4; 3 male, 1 female, 10 mg/kg: *n* = 4; 2/sex], and oxycodone [40 mg/kg: *n* = 3; 2 male, 1 female, 10 mg/kg: *n* = 4; 2/sex]). These opioids were tested using the same study design as was followed for fentanyl. All opioids were administered IM and the respective dosing days were randomized to Days 3, 6 and 9 post-mAb.

### NHP antinociception and operant responding apparatus

During concurrent warm-water tail-withdrawal and operant responding experiments, monkeys were seated in customized Plexiglas chairs that allowed their tails to hang freely behind the chair. While seated, monkeys faced the chair’s front panel which was equipped with colored stimulus lights, two response levers, and a receptacle between each lever into which 30% sweetened condensed milk (0.15 ml) could be delivered. Responding on the active lever (left) with a force of ≥0.2 N produced an audible click and was recorded as a response; responses on the right lever had no scheduled consequences. For all operant studies, a commercially available interface/computer program was used to program schedule contingencies and record data (MED-PC v4, Med Associates Inc., VT, USA).

### NHP antinociception and operant responding procedure

This study was completed in three phases which included the determination of: (1) the baseline fentanyl and other opioids antinociceptive and response-rate decreasing effects; (2) the mitigation of the fentanyl’s effects on tail-withdrawal and operant responding by pre-treatment with CSX-1004; and (3) the selectivity of CSX-1004 by evaluating its effects on tail-withdrawal and response-rates produced by the three other tested opioids.

Tail withdrawal latencies and food-maintained behavior were assessed as described previously^56^. Animals were trained to respond under a fixed-ratio 10-response (FR10) schedule of food reinforcement in the presence of red stimulus lights. Completion of 10 responses in less than 20 s resulted in milk delivery and initiated a timeout (TO) period of 30 s during which all stimulus lights remained off. Failure to complete 10 responses within 20 s initiated the 30 s TO. Tail withdrawal latencies were measured during the 30 s TO periods by immersing the subject’s tail in water held at 35 °C or 52 °C (each temperature of water was presented in a randomized order). Experimental sessions were 4 sequential cycles, each composed of a 10 min TO during which no lights were on and responding had no programmed consequences followed by a 450 s response component during which the FR10 schedule of food reinforcement and interspersed determinations of tail withdrawal latencies was in effect. Cumulative doses of drug were administered at the onset of the 10 min TO. The effects of saline and a wide range of doses of fentanyl (0.00032–0.01 mg/kg), alfentanil (0.0032–0.056), oxycodone (0.032–0.56 mg/kg), and morphine (0.1–1.8 mg/kg) on tail withdrawal latency and food-maintained operant behavior were determined prior to administration of CSX-1004.

Once baseline measurements were established, either 10 (*n* = 4; 2 male, 2 female) or 40 mg/kg (*n* = 4; 3 male, 1 female) CSX-1004 was administered on day 0 as described for the ventilation studies. The animals were then retested 30 min after infusion using the same procedure as described above, receiving 4 saline injections throughout the session. The following day, day 1, the fentanyl dose-response curve was determined at doses of 0.001–0.032 mg/kg for the 10 mg/kg group and at 0.0032–0.1 mg/kg for the 40 mg/kg group. The other three opioids (oxycodone: 0.032–0.56 mg/kg, alfentanil: 0.0032–0.056 mg/kg, and morphine: 0.1–1.8 mg/kg) were tested in random order between days 3–10, always allowing for a training session with no drug administration between testing days to ensure reliable performance on the operant task throughout the study.

### ELISA method for quantifying CSX-1004 in NHPs

Corning 3690 half-area, 96-well microtiter plates were coated with 12.5 ng fentanyl-BSA antigen in water per well and then dried overnight at 37 °C. After blocking in 3% BSA in PBS-T, six 1:2 serial dilutions of each serum sample starting at 1:1200 were run alongside six 1:2 dilutions of CSX-1004 antibody in duplicate starting at 100 ng/mL as a standard curve. Serum samples and CSX-1004 antibody were both diluted in 1% BSA in PBS-T as was the secondary antibody. After a 1 h incubation at 37 °C, plates were washed 5 times with PBS-T, and 1:10,000 goat anti-human IgG HRP (1 mg/mL, Southern Biotech #204005, Birmingham, AL) was added and incubated for 0.5 h at 37 °C. After washing 5 times with PBS-T, TMB substrate (Thermo Fisher #34021, Waltham, MA) was added and plates were stopped using 2 M sulfuric acid and then read at 450 nm using a SpectraMax i3x plate reader running SoftMax Pro version 7.0 software.

### Data analysis and statistics

A within-subject design was used, in which each subject served as its own control and received all relevant test and control conditions. This design permits scientifically meaningful results to be obtained with fewer animals than would be required using other designs. In respiration studies, data from the last 3 min of air or 5% CO_2_ mixtures was used for analysis of drug effects on ventilation. Minute volume (V_E_) was calculated by multiplying the frequency of ventilation (f) (respiration rate) by the tidal volume (V_T_) [V_E_ = V_T_ X f]. The V_E_ data was normalized to within-subject saline values and expressed as a percentage of the saline value (% Saline = V_E test_/V_E saline_ X 100). Similarly, within-subject normalization was performed for antinociception (10 s cutoff = 100% and the saline response = 0%) and operant responding (saline response = 100% and 0 = 0%). For comparisons of potency, ED_50_ values and relative potencies were determined as group means ± SD before and after mAb treatment from data using linear regression analysis of the dose-effect curves without any exclusion of data points. All data was examined at individual and group levels of analysis using GraphPad Prism 8. When ANOVA (one-way or two-way RM) was used, Tukey’s, Dunnett’s, or Bonferroni’s post hoc test for comparison to pre-mAb baseline data was conducted with significance set at *p* < 0.05, 0.01, and 0.001. Data points on graphs are expressed as group means ± SEM. No significant gender-related differences in individual subjects were detected for all rat and NHP experiments; therefore, results containing both genders were not disaggregated.

ELISA data were analyzed using GraphPad PRISM 8 using a 4-PL fit of the CSX-1004 standards to determine unknown serum concentrations of CSX-1004. At least 2-3 dilutions per sample in the working range of the standard curve were used for interpolation and averaged to obtain the values as presented. PK analysis of mAb serum concentrations was performed in PKAnalix version 2021R2 or Phoenix WinNonlin version 8.3 to obtain the PK parameters.

### Reporting summary

Further information on research design is available in the Nature Portfolio Reporting Summary linked to this article.

### Supplementary information

Supplementary Information

Description of Additional Supplementary Files

Supplementary Data 1

Reporting Summary

Peer Review

### Source data

Source Data

## Data Availability

All data supporting the findings of this study are available within the paper and its Supplementary Information. Antibody material is available upon request from the corresponding author. Source data are provided with this paper. The raw data generated in this study have been deposited in the Figshare database under the following address 10.6084/m9.figshare.24354346. Source data are provided with this paper.
