# Supplementary material for: Investigation of monoclonal antibody CSX-1004 for fentanyl overdose
Source: Nat Commun. 2023 Dec 5;14:7700. doi: 10.1038/s41467-023-43126-0 (PMC10698161; doi:10.1038/s41467-023-43126-0)
Supplement: Supplementary file 1 — Supplementary Information [file 41467_2023_43126_MOESM1_ESM.pdf]

Supplementary Information:

Investigation of Monoclonal Antibody CSX-1004 for Fentanyl Overdose

Paul T. Bremer, Emily L. Burke, Andrew C. Barrett, Rajeev I. Desai
Corresponding author: pbremer@cessationtx.com

Contents Include:

Supplementary Methods
Figs. S1 to S5
Tables S1 to S4

Supplementary Methods

LCMS analysis of fentanyl

Blood samples were collected retroorbitally from n = 4 mice from the prevention experiment 48 h after CSX-1004 administration (46 mg/kg, IP) and 20 min after fentanyl administration (0.4 mg/kg, IP). For sample preparation, 60 µL whole blood was pipetted into a new tube along with 8 µL of 50 ng/mL fentanyl-d5 in MeOH. After vortexing, 120 uL of 50 mM K_2CO_3 solution and 420 µL of 7:3 hexane/ethyl acetate were added. The samples were vortexed for 15 seconds and centrifuged at 1000 x g for 5 min. The top solvent layers were pipetted into new tubes and evaporated by Genevac for 1 h. The resulting residues were dissolved in 68 µL MeOH and analyzed on an LC/MS/MS instrument (see below). Standards were prepared in the same manner by extracting blood spiked with fentanyl ranging in concentration from 5000 ng/mL to 1.6 ng/mL. The limit of detection was determined to be around 1.6 ng/mL.

All samples were run on a Waters (Milford, MA) Xevo TQ-XS triple quadrupole instrument with MassLynx v4.1 software.

For fentanyl, 337.1 → 187.99 was the quantitative transition state, and m/z 337.1 → 104.91 was the qualitative transition. For the d5 internal standard, m/z 342.1 → 188 was used.

Fentanyl MS parameters:

337.1 → 187.99, CV = 78 V, CE = 18 V

337.1 → 104.91, CV = 78 V, CE = 28 V

342.1 → 188, CV = 16 V, CE = 24 V

LC conditions were the same for all compounds. The column was a Waters 21 x 50 mm BEH C18, UPLC column with a 1.7 µm particle size. For the mobile phase, water/0.1% formic (A) and acetonitrile/0.1% formic (B) were used. Flowrate was 0.3 mL/min and 5 µL sample was injected. The LC method was as follows:

T = 0 min, 90:10 (A:B)

T = 5 min, 5:96 (A:B)

T = 8 min, 5:95 (A:B)

T = 8.5 min, 90:10 (A:B)

T = 12 min, stop.

ELISA analysis of CSX-1004 in mice

Corning 3690 half-area, 96-well microtiter plates were coated with 25 ng fentanyl-BSA antigen in PBS per well then dried overnight at 37 °C. After blocking in 5% skim milk, six 1:1 serial dilutions of each serum sample starting at 1:1000 were run alongside twelve serial dilutions of CSX-1004 antibody (100-0.05 ng/mL) as a standard curve. Serum samples and CSX-1004 antibody were both diluted in 1% BSA in PBS as was the secondary antibody. After a 2 h incubation at 37 °C in a moist chamber, plates were washed 10 times with water, and 1:10,000 goat anti-human IgG Fc Specific-HRP (1 mg/mL Sigma A-0170) was added and incubated for 1 h as described above. After washing 10 times with water, TMB (Thermo Fisher, Waltham, MA) was added and plates were stopped using 2M sulfuric acid then read at 450 nm. Data were analyzed using GraphPad PRISM 8 using a linear fit of the CSX-1004 standards to determine unknown serum concentrations of CSX-1004. At least 3 dilutions per sample in the linear range of the standard curve were used for interpolation and averaged to obtain the values as presented.

Prevention of fentanyl antinociception

CSX-1004 was tested for its ability to prevent the effects of fentanyl in the tail-flick antinociception test. CSX-1004 (46 mg/kg in PBS, IP) or IP saline was administered to n = 4 mice/group at time 0, and mice were challenged with fentanyl (0.4 mg/kg, IP) at 4 h and 48 h following CSX-1004 administration.

GLP Rat Study

Animals and Animal Maintenance

A. Receipt and Quarantine: A total of 242 Sprague-Dawley CD® IGS rats [Crl:CD(SD)] (121 male and 121 female) were obtained from the Raleigh, NC, facility of Charles River Laboratories, Inc. (Wilmington, MA). The rats were received at IITRI on March 2, 2022. The rats were approximately six weeks old at arrival. One day after arrival, a random sample of 20 male rats weighed 101-157 g, and a random sample of 20 female rats weighed 110-152 g. During the quarantine period (14 days), all rats were observed at least twice daily for evidence of moribundity. A physical examination was also performed on all rats during the quarantine period to ensure their health and suitability as test subjects. The rats were released from quarantine by IITRI's Veterinarian on March 15, 2022.
   Study rats were approximately eight weeks old and weighed 192-263 g (males) and 174-226 g (females) at the initiation of dosing.
B. Group Assignment and Identification: Study rats were assorted into groups using a computerized randomization procedure based on body weights on March 15, 2022, using the Ascentos™ system. Study rats were randomized into groups. Animals selected for the study were assigned a unique identification number following randomization. Each rat was identified by an ear tag. All cages were identified by a card indicating the project number, animal number, dose group and sex. Cage cards were also color-coded according to the study group.
C. Housing and Environment: Upon receipt, rats were group-housed in plastic shoebox cages (up to two animals of the same sex per cage) lined with absorbent hardwood chip bedding and equipped with automatic watering systems in order to acclimate the animals to the watering systems. At the end of the acclimation period and for the remainder of the study, rats were individually housed in plastic shoe-box cages with automatic watering systems. The temperature and relative humidity (RH) of the animal room were recorded daily and generally met the conditions specified in the applicable IITRI Standard Operating Procedure (20-26º C and 30-70% RH). Fluorescent lighting in the animal room was provided on a cycle of 12 hours of light followed by 12 hours of darkness.
D. Food and Water: Rats were provided with certified Teklad Global 14% Protein Rodent Diet [2014C; Envigo (Indianapolis, IN)]. Each certified lot of diet was analyzed for contaminants to ensure that none are present at concentrations that would be expected to interfere with the conduct or purpose of this study. Analytical data from lots of diet used in this study are retained on file at IITRI.
   Coarse-filtered City of Chicago water was provided *ad libitum* by means of an in-cage automatic watering system. No contaminants were known to be present in the water at levels that would be expected to interfere with the conduct or outcome of the study. Reports for the food and water analyses are maintained with IITRI's facility records.
E. Animal Welfare: Procedures for animal care and housing complied with all applicable sections of the Animal Welfare Act (Title 9, *Code of Federal Regulations*), the Public Health

Service Policy on Humane Care and Use of Laboratory Animals (NIH Office of Laboratory Animal Welfare, 2015) and the *Guide for the Care and Use of Laboratory Animals* (National Research Council, 2011).

F. Experimental Design: The experimental design for this study was as follows:

Group	CSX-1004 Dose Level (mg/kg)	Dose Volume (mL/kg)	Number of Rats					
			Main Study		Recovery		Toxicokinetic	
			Male	Female	Male	Female	Male	Female
1	0 (Vehicle Control)	10	15	15	5	5	3	3
2	10	10	15	15	5	5	6	6
3	100	10	15	15	5	5	6	6
4	400	10	15	15	5	5	6	6

Dosing was initiated on March 15, 2022 (Study Day 1) for all Main Study and Recovery males. Dosing was initiated on March 16, 2022 (Study Day 1) for Main Study and Recovery females. Dosing was initiated March 17, 2022, for all Toxicokinetic (TK) rats. Scheduled terminal necropsies occurred on Study Day 66 (i.e., May 19, 2022, for Main Study males and May 20, 2022, for Main Study females). Scheduled recovery necropsies occurred on Study Day 92 (i.e., June 14, 2022, for Recovery males and June 15, 2022, for Recovery females). Scheduled TK necropsies occurred on Study Day 93 (June 17, 2022).

G. Toxicology Methods

1. Administration: Dose formulations were administered to each study rat by intravenous (IV) slow bolus injection (lasting approximately 30 seconds) via the tail vein on Days 1, 22, 43 and 64. The dose was calculated by Ascentos™ using each rat's most recent body weight and a dosing volume of 10 mL/kg body weight.

2. Mortality/Moribundity, Clinical Observations and Physical Examinations: All surviving rats were observed at least twice daily during the quarantine, treatment, and recovery periods for mortality or evidence of moribundity. Any abnormal clinical signs were recorded. Moribundity/mortality checks were separated by a minimum of four hours.

Clinical observations (cage-side observations) were conducted on Main Study and Recovery rats daily throughout the treatment period and recovery periods. Physical examinations (detailed hand-held clinical observations) were conducted on each rat once during quarantine, on each surviving Main Study and Recovery rat on every dosing day at 1 to 2 hours post-dose, and on each surviving Main Study and Recovery rat weekly throughout the treatment and recovery periods.

3. Body Weights: All rats were weighed at randomization, and all study rats were weighed at least once weekly throughout the treatment and recovery periods. Fasted body weights were collected from all surviving Main Study and Recovery rats on the day prior to scheduled necropsy.

4. Food Consumption: Food consumption was measured individually for all surviving Main Study and Recovery rats at least once weekly throughout the treatment and recovery periods.

5. Ophthalmic Examinations: Indirect funduscopic examinations were performed by a board-certified veterinary ophthalmologist on all rats during pre-test and on all surviving Main Study and Recovery rats during Study Week 9 or 10.

6. Clinical Pathology: Blood samples for clinical chemistry and hematology determinations were collected on Study Days 66 and 92. Rats were fasted overnight prior to blood collection. Prior to blood collection, each rat was lightly anesthetized with 70% CO_2 / 30% air. Blood was collected from all surviving Main Study and Recovery rats for clinical chemistry and hematology via puncture of the retro-orbital plexus. Rats scheduled for necropsy (i.e., Main Study rats on Study Day 66 and Recovery rats on Study Day 92) were then anesthetized with an injection of sodium pentobarbital, and a blood sample for coagulation was collected from the abdominal aorta.

Urine samples were collected from all Main Study and Recovery rats on Study Day 66 and from Recovery rats on Study Day 92. For urine collection, rats were individually housed in metabolism cages overnight. Urine was collected from a pan below each cage.

7. Immunogenicity Analysis: Blood samples for determination of anti-CSX-1004 antibodies (via anti-drug antibody ELISA) were collected from three TK rats/sex in Group 1 and from six TK rats/sex in Groups 2-4 prior to dosing on Days 1, 22, 43, and 64. Blood samples were also collected from three TK rats/sex in Groups 2-4 on Day 85.

Immunogenicity blood samples were collected into serum-gel clotting activator tubes and allowed to clot. Blood samples were then centrifuged, and the resulting serum was aliquoted into storage tubes. Serum samples were stored frozen at ≤ -65°C until analyzed at IITRI.

8. Serum Drug Level Analysis: Blood samples (approximately 0.4 to 0.5 mL per time point) for serum drug level analysis (via ELISA) were collected from three TK rats/sex in Groups 2-4 after dosing on Days 1 and 64 at the following time points: 0.083, 1, 4, 8, 24, 48, 96, 144, 240, 288, 408, and 504 (the last sample following the first dose was collected on Day 22, prior to administration of the second dose) hours post dose. Blood samples were also collected from three TK rats/sex in Group 1 at 1 hour post-dose on Days 1 and 64. Prior to blood collection via puncture of the retro-orbital plexus, each study rat was lightly anesthetized using 70% CO_2/30% air or 70% CO_2/30% O_2. No TK animal was bled any more than three times over a 24-hour period.

Serum drug level blood samples were collected into serum-gel clotting activator tubes and allowed to clot. Blood samples were then centrifuged, and the resulting serum was aliquoted into storage tubes. Serum samples were stored frozen at ≤ -70°C until analyzed at IITRI.

9. Toxicokinetic (TK) Analysis: Toxicokinetic calculations were performed using serum drug ELISA level data and composite concentration-time profiles of CSX-1004 in rat serum using Phoenix WinNonlin software [version 8.3; Certara (Princeton, NJ)]. Reported results were calculated using PKAnalix version 2021 and averaged across all rats in each dose cohort. Values differed from WinNonlin calculations by <10%.

10. Euthanasia and Necropsy: On Study Day 66 (two days after the last dose), all Main Study rats were euthanized and received a complete necropsy. On Study Day 92, all Recovery rats were euthanized and received a complete necropsy. Prior to scheduled necropsy, rats were fasted overnight. After being anesthetized with 70% CO_2/30% air for blood collection, rats were euthanized by exsanguination. Necropsy consisted of examination of the external surface of the body, all orifices, and the cranial, thoracic and peritoneal cavities and their contents. All surviving TK rats were euthanized after the last scheduled blood collection and discarded without necropsy.

The tissues listed below were collected and fixed in 10% neutral buffered formalin, with the exceptions of the eyes (with optic nerves) and Harderian glands, which were fixed with Davidson's solution; the testes and epididymides, which were fixed with modified Davidson's solution; and the bone marrow smear, which was fixed in methanol. Select tissues (marked with an asterisk in the following list) were weighed (paired organs weighed together) and organ-to-body weight ratios were calculated using the fasted body weight for each animal.

Administration site[1]	Gland, seminal vesicle (paired)	Small intestine, ileum
Animal identification[2]	Gland, thyroid (paired)[3]	Small intestine, jejunum
Artery, aorta	Heart*	Skin, ventral abdomen
Bone, femur	Kidney (paired)*	Spinal cord, cervical
Bone, sternum	Large intestine, cecum	Spinal cord, lumbar
Bone marrow, femur	Large intestine, colon	Spinal cord, thoracic
Bone marrow, sternum	Large intestine, rectum	Spleen*
Brain*	Liver*	Stomach
Epididymis (paired)	Lung	Testis (paired)*
Esophagus	Lymph node, mandibular	Thymus*
Eye (paired)	Lymph node, mesenteric	Trachea
Gland, adrenal (paired)*	Muscle, skeletal	Urinary bladder
Gland, Harderian (paired)	Nerve, optic (paired)	Uterus
Gland, mammary (females)	Nerve, sciatic	Vagina
Gland, parathyroid (paired)[3]	Ovary (paired)*	Bone marrow smear (femur)[4]
Gland, pituitary	Pancreas	Gross lesions, masses, abnormal tissues (if any)
Gland, prostate	Small intestine, duodenum	
Gland, salivary (paired)		

* Weighed at scheduled necropsies only.
[1] IV injection site was the tail vein.
[2] Metal ear tag or tail marking; collected but not processed.
[3] Due to size, this organ was only evaluated if present in normal section.
[4] Bone marrow smears were prepared at scheduled necropsy only.

11. Histopathology: The tissues and organs listed above (except for the animal identification and bone marrow smears) collected at terminal necropsy from all Main Study rats in Groups 1 and 4 were evaluated microscopically. Additionally, injection sites and all gross lesions were evaluated microscopically in the low and mid dose Day 66 animals and all recovery euthanasia animals. No target organs were identified following microscopic evaluation of the high-dose tissues at the terminal necropsy. All evaluations were performed by a board-certified veterinary pathologist.

Tissues to be examined microscopically were trimmed, processed routinely, embedded in paraffin and stained with hematoxylin and eosin.

G. <u>Statistical Methods</u>: Descriptive statistics (mean and standard deviation) were calculated and analyzed for statistical significance for the following data using the Ascentos™ system:

- Body weights and body weight changes
- Clinical pathology [clinical chemistry, hematology and coagulation parameters and select urinalysis parameters (volume, pH, refractive index, specific gravity, sodium, potassium, chloride and osmolality)]
- Organ weights and organ-to-body weight ratios

For all analyses, if the data set was normally distributed and of equal variance, statistical comparisons were conducted using a one-way analysis of variance (ANOVA), with *post hoc* comparisons made (if necessary) using Dunnett's test. If normality and/or equal variance failed for a data set, statistical comparisons were conducted using nonparametric Kruskal-Wallis ANOVA, with post hoc comparisons made (if necessary) using Dunn's test. Incidence data (*i.e.*, clinical signs, physical examination observations and fate data) were evaluated using Chi-square analysis and/or Fisher's Exact test. A minimum significance level of $p < 0.05$ was used for the statistical comparisons in this study.

RESULTS

A. <u>Dose Formulation Analysis</u>:
Measured concentrations (determined in either duplicate or quadruplicate) were within acceptable limits (90-110% of the target values), with accuracy ranging from 91% to 102%, with the exception of formulations prepared at 1.0 mg/mL, which had accuracy values of 80% (Study Day 22 preparation) and 69% (Study Day 64 preparation).

B. <u>Mortality and Clinical Signs</u>:
No test article-related mortality was seen in any group during the course of this study. The following clinical signs were observed during weekly physical examinations:
Alopecia: one Group 2 (10 mg/kg) male
Swelling (due to blood collection in the right eye): one Group 2 female
Scab: one Group 2 and one Group 3 (100 mg/kg) female
None of the clinical signs seen during this study were considered test article-related.

C. <u>Body Weight and Body Weight Change</u>:
No statistically significant differences in body weight or decreases in body weight change in comparison to Group 1 (Vehicle Control) rats of the same sex were seen in the test article-related groups during this study.

D. <u>Food Consumption</u>:
A statically significant decrease of 9% in food consumption in comparison to Group 1 (Vehicle Control) animals of the same sex were seen in Group 3 (100 mg/kg) females at the Day 43 to 50 interval. The decrease in food consumption was not considered treatment-related since it was only seen in one sex and at one interval and was not seen in the high dose group.

E. <u>Ophthalmic Examination</u>:
No test article-related ocular abnormalities were seen in either sex during the pre-test or at Study Week 9 examinations.

F. <u>Clinical Pathology</u>:
No toxicologically relevant or test article-related changes were seen in any clinical pathology (clinical chemistry, hematology, coagulation, and urinalysis) parameter at Study Days 66 or 92.

G. <u>Immunogenicity Analysis</u>:

Only two serum samples [both from the same animal – Group 3 (100 mg/kg) female C185 at Days 64 and 85] from all groups and time points assayed screened positive for anti-CSX-1004 antibodies.

H. Serum Drug Level Analysis:

All serum CSX-1004 levels for Group 1 (Vehicle Control) rats were below the standard curve of the assay (<1.6 ng/mL). For Groups 2-4, the serum ELISA results were used to determine the toxicokinetics for the test article.

I. Toxicokinetics:

After IV administration of doses of 10 to 400 mg/kg/dose of CSX-1004, median tmax was 0.08 hours (range of 0.08 to 4 hours). Overall mean t1/2 of CSX-1004 was 187 hours (range of 119 to 249 hours). Mean CL was 0.21 mL/hr/kg (range of 0.12 to 0.29 mL/hr/kg) and mean Vz was 56.0 mL/kg (range of 30.7 to 87.1 mL/kg). Systemic exposure (evaluated as Cmax, AUClast and AUCINF) in females was slightly greater than in males on Day 1, but on Day 64 exposure was similar in females and males. There was no consistent relationship for systemic exposure between Day 1 and Day 64. Systemic exposure increased as dose increased, although the relationship to dose was not always proportional to dose.

J. Organ Weights:

No statistically significant changes in absolute organ weight were observed at the end of the treatment or recovery periods. Statistically significant decreases in relative organ weights (organ-to-body weight ratios) were observed in the liver and kidney of Group 2 (10 mg/kg) males at the end of the treatment period. However, these changes were not considered test article-related since they were only seen in one sex and there was no dose-response relationship. At the end of the recovery, no statistically significant differences in relative organ weights were seen in any test article-treated group.

J. Necropsy and Histopathology:

Intravenous slow bolus injection of CSX-1004 via the tail vein of rats once every three weeks for a total of four injections at doses of 0, 10, 100, 400 mg/kg/dose resulted in no early deaths. There were no CSX-1004 related macroscopic or microscopic findings attributable to CSX-1004 administration in either the terminal or recovery groups.

DISCUSSION AND CONCLUSION

Following intravenous (IV) slow bolus administration of CSX-1004, a monoclonal antibody, to rats at dose levels of 0, 10, 100, or 400 mg/kg on Study Days 1, 22, 43 and 64, no test article-related mortality, clinical signs of toxicity, or effects on body weight or food consumption were seen. No test article-related ophthalmic lesions were seen, and no toxicologically relevant or test article-related changes were seen in clinical pathology parameters at the end of the treatment and recovery periods. Only one rat (a female dosed at 100 mg/kg) screened positive (at Study Days 64 and 85) for serum anti-CSX-1004 antibodies via ADA ELISA. At the end of the treatment and recovery periods, no test article-related effects on absolute or relative organ weights were seen. In addition, no test article-related gross or microscopic findings were noted in any test article-treated group.

In conclusion, the No Observed Adverse Effect Level (NOAEL) in rats following four IV slow bolus administrations (on Study Days 1, 22, 43, and 64) of CSX-1004 at dose levels of 0, 10, 100 or 400 mg/kg was 400 mg/kg based on the lack of systemic toxicity at this level.

ELISA Method for Quantifying CSX-1004 Antibodies Against Fentanyl-BSA Antigen: A validated ELISA method was used to detect and quantify CSX-1004 (human IgG) antibodies against Fentanyl-BSA antigen in rat serum samples as described below:

1. ELISA Plate Coating: Plate Coating Solution was prepared with Fen-BSA antigen (provided by the Sponsor). The solution was prepared by diluting the antigen in 1X PBS Coating Buffer to a concentration of 0.5 µg/mL. Microtiter plates were coated with 50 µL per well of the appropriate 1X PBS Coating Buffer. Wells were visually inspected to ensure equal coating of the wells. The plates were then left uncovered and incubated overnight in a controlled incubator at 35-39°C.
2. Plate Blocking: Plates were visually inspected to ensure the wells had completely evaporated. Next, the plates were allowed to equilibrate to room temperatures for 5-10 minutes prior to continuing. The dried-out plates were then blocked with 50 µL of 3% BSA Blocking Buffer per well. The plates were incubated with the Blocking Buffer for at least 30 minutes ± 2 minutes at 35-39°C).
3. Sample and Standard Preparation: All standard and serum samples were thawed and equilibrated to room temperature prior to being diluted with Assay Diluent. Stock standards were thawed and diluted 1:40 in assay diluent on the day of the assay. A standard curve ranging from 100-1.6 ng/mL with 2-fold dilution steps was prepared. The positive control used for the standard curve was CSX-1004 (provided by the Sponsor).
4. Sample and Standard Addition: After being incubated for 30 ± 2 minutes with Blocking Buffer, the plates were inverted and then blotted with a paper towel and 50 µL of each standard, assay blank (Assay Diluent), and diluted sample was loaded into the appropriate wells. All wells were visually inspected to ensure even distribution within the wells. The plates were then covered and incubated at 35-39°C for 60 ± 2 minutes.
5. HRP-Conjugate Preparation and Addition: An HRP-conjugate Solution was prepared by serially diluting the Goat anti-Human IgG HRP-conjugate (Sigma; St. Louis, MO) to a 1:10,000 dilution in Assay Diluent. After being incubated 60 ± 2 minutes with the standards, positive control, assay blank and samples, the plates were washed five times with approximately 350 µL of wash buffer per well using a BioTek 405 LS or a BioTek ELx405 plate washer. The washed plates were blotted with a paper towel and 25 µL of the prepared HRP-conjugate Solution was added to each well. The wells were visually inspected to ensure that the HRP-conjugate Solution was evenly distributed in the well. The plates were then covered and incubated for 30 ± 2 minutes at 35-39°C.
6. Substrate Preparation and Addition: Within 15 minutes of use, equal volumes of the substrate system components were mixed to create the TMB Substrate Solution.
   After being incubated 30 ± 2 minutes with the HRP-conjugate Solution, the plates were then washed five times with approximately 350 µL of Wash Buffer per well using a BioTek 405 LS or BioTek ELx405 plate washer. The washed plates were blotted with a paper towel and 50 µL of TMB Substrate Solution was added to each well. The wells were visually inspected to verify the even distribution of the solution within the wells. The plates were incubated with the Substrate Solution for 30 ± 2 minutes at room temperature on a plate shaker set at 150 RPM.
   Following incubation with Substrate Solution, 50 µL of Stop Solution was added to each well. The plates were gently tapped or placed on a plate shaker for 1-3 minutes to ensure the even distribution of the stop solution.
7. Plate Reading: Plates were read using a 450 nm filter on a Molecular Devices plate reader with SoftMax Pro software (version 7.0.3; GXP; San Jose, CA). The results were calculated using a four-parameter logistic function (4-PL). The standard curve fit was based upon the following equation: Y=D+((A-D)/(1+(X/C)^B)), where the coefficient C is the x-value corresponding to the y-value that is the midpoint between A (the y-value corresponding to the asymptote at low values of the x-axis) and D (the y-value corresponding to the asymptote at high values of the x-

axis), and the coefficient B describes how rapidly the curve makes its transition from the asymptotes in the center of the curve (i.e., the slope). The sample results were then interpolated. The reported result of each sample was the adjusted mean result of the replicates for the lowest dilution within the range of the Standard Calibration curve. The adjustment accounts for the dilution of the reported mean value. Sample results that were below the standard curve of the assay were reported as "<SSV". The lower limit of detection for this assay was 1.6 ng/mL.

8. Acceptance Criteria: The assay acceptance criteria are listed below.
    A. Calibration Standards must have the following OD value precision:
        - ≤ 15% CV between replicates of the 3.13, 6.25, 12.5, 25, and 50 ng/mL calibrators
        - ≤ 20% CV between replicates for the 1.6 and 100 ng/mL calibrators
    B. A calibration curve must include at least five acceptable Calibration Standard concentrations. The seven working Calibration Standard concentrations are 1.6, 3.13, 6.25, 12.5, 25, 50, and 100 ng/mL. If three or more of these seven calibrators do not meet the acceptable CV values, the plate will be retested.
    C. The square of the coefficient of correlation (r^2) of the Calibration Standard curve must be ≥ 0.95.
    D. The OD_{450} value of the blank wells must be ≤ 0.094.
        - The mean response of each reported value must be within the dynamic range of the standard curve as defined by the highest and lowest values of the standard curve.

ADA ELISA Method for Quantifying anti-CSX-1004 Antibodies: The following validated ADA ELISA method was used to detect and quantify anti-CSX-1004 antibodies in the rat serum samples:

1. ELISA Plate Coating: Plate Coating Solution was prepared with Fen-BSA antigen (provided by the sponsor). The solution was prepared by diluting the antigen in 1X PBS Coating Buffer to a concentration of 0.5 µg/mL. Microtiter plates were coated with 50 µL per well of the appropriate 1X PBS Coating Buffer. Wells were visually inspected to ensure equal coating of the wells. The plates were then left uncovered and incubated overnight in a controlled incubator at 35-39°C.
2. Plate Block 1: Plates were visually inspected to ensure the wells had completely evaporated. Next, the plates were allowed to equilibrate to room temperature for 5-10 minutes prior to continuing. The dried-out plates were then blocked with 300 µL of 5% BSA Blocking Buffer per well. The plates were incubated with the Blocking Buffer for at least 30 ± 2 minutes at 35-39°C).
3. Immobilization (Second Coat): After being incubated for 30 minutes ± 2 minutes with Blocking Buffer, the plates were inverted and dabbed against absorbent paper to remove the blocking buffer. Then 50 µL of CSX-1004 diluted to 1,000 ng/mL in assay diluent was added to each well. The plates were covered and incubated at 37°C ± 2°C for 60 ± 2 minutes.
4. Plate Block 2: After being incubated for 60 ± 2 minutes with CSX-1004, the plates were washed three times with approximately 350 µL of Wash Buffer per well. The washed plates were then blotted with a paper towel and 300 µL of 5% BSA Blocking Buffer was added to each well. The plates were then covered and incubated for at least 2 hours at 35-39°C.
5. Sample and Standard Preparation: All Quality Controls and serum samples were thawed and equilibrated to room temperature prior to being diluted with Assay Diluent. Quality Controls were prepared by diluting stocks 1:40 on the day of the assay. Quality Controls after dilution were 300, 100, and 33.3 ng/mL. The positive control used for the Quality Controls was purified rat anti-human Fc (BioLegend; lot 410701). Screening assay serum samples were

diluted 1:80 in assay diluent. Confirmatory assay serum samples were diluted 1:40 and then mixed 1:1 with 6000 µg/mL of CSX-1004 in assay diluent. Confirmatory assay samples were incubated for 1-4 hours ± 5 minutes in a round bottom plate shaking at 150 RPM (for a final sample dilution of 1:80). Quality Controls for the confirmatory assay were diluted 1:20 and then 1:1 with the CSX-1004.

6. Sample and Standard Addition: After being incubated for 30 ± 2 minutes with Blocking Buffer, the plates were washed three times with approximately 350 µL of Wash Buffer per well using a BioTek 405LS or a BioTek ELx405 plate washer. The washed plates were then blotted with a paper towel and 50 µL of each standard, assay blank (Assay Diluent), and diluted sample was loaded into the appropriate wells. All wells were visually inspected to ensure even distribution within the wells. The plates were then covered and incubated at 35-39°C for 60 ± 2 minutes.
7. HRP-Conjugate Preparation and Addition: An HRP-conjugate Solution was prepared by serially diluting the Goat anti-Rat IgG HRP-conjugate (Abcam) to a 1:70,000 dilution in Assay Diluent. After being incubated 60 ± 2 minutes with the standards, positive control, assay blank and samples, the plates were washed three times with approximately 350 µL of wash buffer per well using a BioTek 405 LS or a BioTek ELx405 plate washer. The washed plates were blotted with a paper towel and 50 µL of the prepared HRP-conjugate Solution was added to each well. The wells were visually inspected to ensure that the HRP-conjugate Solution was evenly distributed in the well. The plates were then covered and incubated for 30 ± 2 minutes at 35-39°C.
8. Substrate Preparation and Addition: Within 15 minutes of use, equal volumes of the substrate system components were mixed to create Substrate Solution.
   After being incubated for 30 ± 2 minutes with the HRP-conjugate Solution, the plates were then washed five times with approximately 350 µL of Wash Buffer per well using a BioTek 405 LS or BioTek ELx405 plate washer. The washed plates were blotted with a paper towel and 100 µL of Substrate Solution was added to each well. The wells were visually inspected to verify the even distribution of the solution within the wells. The plates were incubated with the substrate solution for 5 ± 2 minutes at room temperature on a plate shaker set at 150 RPM.
   Following incubation with the Substrate Solution, 100 µL of Stop Solution was added to each well. The plates were gently tapped or placed on a plate shaker for 1-3 minutes to ensure the even distribution of the stop solution.
9. Plate Reading: Plates were read using a 450 nm and 650 nm filter on a Molecular Devices plate reader with SoftMax Pro software (version 7.0.3; GXP; San Jose, CA). Wavelength correction was performed by subtracting the OD_{650} value from the OD_{450} value (Adjusted OD_{450}). The reported result of each sample is the Adjusted OD_{450}.
10. Acceptance Criteria: The assay acceptance criteria are listed below.
    A. Calibration Standards must have the following OD value precision:
       - OD_{450} precision of ≤ 20% CV between replicates of the negative control (NQC), 33.3 (LQC), 100 (MQC), and 300 (HQC) ng/mL QCs
       - HQC mean OD_{450} between 0.71 and 1.07
       - MQC mean OD_{450} between 0.58 and 0.86
       - LQC mean OD_{450} between 0.40 and 0.60
       - NQC mean OD_{450} ≤ 0.221
    B. Sample Evaluation Criteria:

- The %CV is ≤ 20% for every sample reported.

C. Screening Assay: ADA Criteria:

- Samples below the Screening Assay Cut-Point (SACP) for their respective plate are negative for ADA and do not need further confirmation testing.
- The SACP is determined by taking the average of the negative control Adjusted OD_{450} on each respective plate and applying the correction factor (CF) of 0.091. The base SACP is 0.282.

D. Confirmatory Assay: ADA Criteria

- % INH for HPC is between 52.6 and 78.9
- % INH for MQC is between 52.8 and 79.1
- % INH for LQC is between 40.4 and 60.6
- Samples below the Confirmatory Assay Cut-Point (CACP) are negative for ADA. The CACP is 70.5% Inhibition.

Human Tissue Cross-Reactivity (TCR)

The objective of this study was to determine the potential cross reactivity of CSX-1004, a monoclonal human IgG1 antibody directed against fentanyl and related synthetic opioids, in cryosections from a full panel of normal human tissues. In order to detect binding, the test article, designated CSX-1004, was applied to cryosections of normal human tissues (at least 3 donors per tissue, as available) at two concentrations (15 and 5 µg/mL). In addition, the test article was substituted with a human IgG1 antibody, which has a different antigenic specificity from that of the test article, designated HuIgG1 (control article). Other controls were produced by omission of the test or control articles from the assay (assay control). CSX-1004 produced weak staining of the positive control material (Fentanyl-BSA antigen UV- resin spot slides [designated Fentanyl-BSA]) at both staining concentrations. CSX-1004 did not specifically react with the negative control material (human hypercalcemia of malignancy peptide, amino acid residues 1-34, UV-resin spot slides [designated PTHrP 1 34]) at either staining concentration. The control article, HuIgG1, did not specifically react with either of the positive or negative control materials. There also was no staining of the assay control slides. The specific reactions of CSX-1004 in all staining runs with the positive control material and the lack of specific reactivity with the negative control material, as well as the lack of reactivity of the control article, indicated that the assay was sensitive, specific, and reproducible.

Binding with CSX-1004 in the human tissue panel was limited to the cytoplasm or cytoplasmic granules of infrequent epithelial cells in the skin (epidermis [stratum corneum]) and uterus (endometrial glands). This binding in both the skin and uterus was cytoplasmic in nature, and binding to cytoplasmic sites in tissue cross reactivity studies generally is considered of little to no toxicologic significance due to the limited ability of antibody drugs to access the cytoplasmic compartment in vivo.

Test Material Identification

	Test Article
**Identification:**	CSX-1004
**Target Antigen:**	Fentanyl and related synthetic opioids
**Structural Category:**	Monoclonal human IgG1 antibody
**Batch/Lot No.:**	211116-0101-BDS
**Testing Facility Tracking No.:**	A50700 or equivalent
**Physical Description:**	Frozen solution
**Purity:**	98.3%, staining calculations will not be corrected for purity
**Concentration:**	100.9 mg/mL
**Storage Conditions:**	Temperature set to maintain -65°C or below; aliquots thawed for single use
**Provided by:**	KBI Biopharma Inc.

Control Material Identification

	Control Article
**Identification:**	Human IgG1 kappa
**Alternate Identification:**	HuIgG1
**Target Antigen:**	None (isotype control for Test Article)
**Structural Category:**	Polyclonal human IgG1κ antibody
**Testing Facility Tracking No.:**	A51163-A51164 or equivalent
**Storage Conditions:**	Temperature set to maintain 2°C to 8°C
**Provided by:**	Abcam (Catalog No. ab206198)

Tissues for Study

- Positive Control: Fentanyl-BSA antigen UV-resin spot slides (**spotted at 40 µg/mL**), protein provided by EastCoast Bio; (Catalog No. LA395) or equivalent, designated Fentanyl-BSA Note: two sets of positive control spot slides were prepared for each run. Staining with CSX-1004 in only one spot slide at each concentration is necessary to accept the run.
- Negative Control: Human hypercalcemia of malignancy peptide, amino acid residues 1-34, UV-resin spot slides (**spotted at 40 µg/mL**), protein provided by Sigma-Aldrich (Catalog No. H9148) or equivalent, designated PTHrP 1-34

Positive and negative control proteins were stored in a freezer set to maintain -30°C to -18°C with thawed material stored in a refrigerator set to maintain 2°C to 8°C.

Human Tissue (Normal) from Three Separate Individuals

Adrenal	Kidney (glomerulus, tubule)	Skin
Bladder (urinary)	Liver	Spinal Cord
Blood Cells [a]	Lung	Spleen
Blood Vessels (endothelium) [b]	Lymph Node	Striated Muscle (skeletal)
Bone Marrow	Ovary	Testis
Brain – cerebellum	Pancreas	Thymus
Brain – cerebral cortex	Parathyroid	Thyroid
Breast (mammary gland)	Peripheral Nerve	Tonsil
Eye	Pituitary	Ureter
Fallopian Tube (oviduct)	Placenta	Uterus – cervix
Gastrointestinal (GI) Tract [c]	Prostate	Uterus – endometrium
Heart	Salivary Gland	

[a] Evaluated from peripheral blood smears or OCT blocks. [b] Evaluated from all tissues where present.
[c] Includes esophagus, large intestine/colon, small intestine, and stomach (including underlying smooth muscle).

Samples (from at least three unique individuals) from each of the above listed tissues were stained and evaluated. Fresh, unfixed tissue samples from the tissue suppliers were/were placed into molds, filled with Tissue-Tek® OCT (Optimal Cutting Temperature) Compound, and frozen. Tissues were stored in a freezer set to maintain -65°C or below until use. Sections were cut at approximately 5 µm. Just prior to staining, the slides were fixed in 10% neutral-buffered formalin (NBF) for 10 seconds at room temperature. Human blood smears were stored in a freezer set to maintain -65°C or below until fixation and staining.

Test Control Material Staining Procedure

Primary Antibody+Secondary Antibody Precomplex	Avidin-Biotin Complex (ABC)	3,3'-Diaminobenzidine (DAB)
Test Article + DkαHuIgG<br>CSX-1004<br>15 and 5 µg/mL	X	X
Control Article + DkαHuIgG<br>HuIgG1<br>15 and 5 µg/mL	X	X
Assay Control (DkαHuIgG)	X	X

Methods for immunohistochemistry were used to eliminate simultaneously the requirement for labeling (e.g., biotin, peroxidase, or fluorescein) of the primary antibody (test or control article) and to preclude nonspecific reactivity between the secondary labeled anti-human IgG and IgG endogenous to the tissues.[1-3]

In this method, the labeled secondary antibody was allowed to attach specifically to the unlabeled primary antibody (either test or control article) by overnight incubation of the primary/secondary antibody mixtures prior to application to the tissue cryosections. The test or control article were mixed with biotinylated $F(ab')_2$ donkey anti-human IgG, Fcγ fragment-specific (DkαHuIgG) antibody at concentrations which achieve a primary:secondary antibody ratio of 1:1.5 on the day prior to staining. Thus, the higher concentration of either test or control article were precomplexed with 22.5 µg/mL of biotinylated DkαHuIgG (Note: the assay control also contains the higher concentration of secondary antibody), while the lower concentration of test or control article were precomplexed with 7.5 µg/mL of biotinylated DkαHuIgG. Precomplexed antibodies were incubated overnight on a rocker mechanism in a refrigerator set to maintain 2°C to 8°C. Prior to use of the antibody on the subsequent day, human gamma globulins were added to each vial to achieve a final concentration of either 4.5 mg/mL (higher concentration of secondary antibody) or 1.5 mg/mL (lower concentration of secondary antibody), and antibodies were incubated for at least 2 hours on the rocker mechanism in a refrigerator set to maintain 2°C to 8°C.

On the day of staining, the slides were rinsed twice with Tris-buffered saline, 0.15M NaCl, pH 7.6 (TBS). Endogenous peroxidase will then be quenched by incubation of the slides with the Dako peroxidase blocking reagent for 5 minutes. Next, the slides were rinsed twice with TBS, incubated with the avidin solution for 15 minutes, rinsed once with TBS, incubated with the biotin solution for 15 minutes, and rinsed once with TBS. The slides will then be treated with a protein block designed to reduce nonspecific binding for 20 minutes. The protein block was prepared as follows: TBS + 1% bovine serum albumin (BSA); 0.5% casein; and

1.5% normal donkey serum. Following the protein block, the precomplexed primary and secondary antibodies were applied to the slides for 2 hours. Next, the slides were rinsed twice with TBS, treated with the ABC Elite reagent for 30 minutes, rinsed twice with TBS, and then treated with DAB for 4 minutes as a substrate for the peroxidase reaction. All slides were rinsed with tap water, counterstained, dehydrated, and mounted.

TBS + 1% BSA served as the diluent for all antibodies and ABC Elite reagent.

Anti-β_2-microglobulin Staining Procedure

Primary Antibody	Secondary Antibody	ABC	DAB
Anti-β_2-microglobulin 1 µg/mL	X	X	X

Sections of each human test tissue were cut at approximately 5 µm. Just prior to staining, the tissues (including blood smears, if utilized) were fixed in 10% neutral-buffered formalin (NBF) for 10 seconds at room temperature.

Fixed cryosections were rinsed twice in phosphate-buffered saline, 0.15 M NaCl, pH 7.2 (PBS). Endogenous peroxidase will then be quenched by incubation of the slides with a solution containing sodium azide (1mM), glucose (10mM), and glucose oxidase (2U/mL) for 1 hour at approximately 35°C. Next, the slides were rinsed twice with PBS, incubated with the avidin solution for 15 minutes, rinsed once with PBS, incubated with the biotin solution for 15 minutes, and rinsed once with PBS. The slides will then be treated with a protein block designed to reduce nonspecific binding for 20 minutes. The protein block was prepared as follows: PBS + 1% bovine serum albumin (BSA); 0.5% casein; and 1.5% normal goat serum. Following the protein block, the anti-β_2-microglobulin antibody was applied to the slides at the concentration listed above for 1 hour. Next, the slides were rinsed twice with PBS, and the biotinylated secondary antibody (goat anti-rabbit IgG) were applied to the slides at a concentration of 2 µg/mL for 30 minutes. Then, the slides were rinsed twice with PBS, reacted for 30 minutes with the ABC Elite reagent, and rinsed twice with PBS. Next, DAB was applied for 4 minutes as a substrate for the peroxidase reaction. All slides were rinsed with tap water, counterstained, dehydrated, and mounted.

PBS + 1% BSA will serve as the diluent for all antibodies and the ABC Elite reagent.

Microscopic Evaluation

After staining, slides were visualized under light microscopy by the IS for Immunopathology. Each slide was judged for adequacy of tissue elements. (M) if there is no tissue present on the slide or if the tissue present is not the expected tissue. Alternatively, a tissue may not be evaluated (NE) if judged inadequate for evaluation (e.g., missing tissue elements, suboptimal morphology). If judged adequate for interpretation by the IS for Immunopathology, each slide (test article, control article, and assay control) will then be examined for the presence of stained cell types or tissue elements. Each stained cell type or tissue element were identified, the subcellular (or extracellular) location of the staining was recorded, and the intensity (strength) of staining were assigned for each slide. Frequency of cell type staining will also be assigned to provide the approximate percentage of cells of that particular cell type or tissue element with staining. UV-resin spot slides were evaluated for intensity only. Tissue comments may also be included for individual control materials and test tissues to provide further description of the tissue and/or staining. The staining intensity and frequency scales to be used for the evaluation of test article, control article, or assay control slides are listed below. All slides stained with the anti-β_2-microglobulin antibody were interpreted as being negative (Neg) or positive (Pos) for staining.

Staining Intensity	
SCORE	RESULT
Neg	Negative (no stained cells)
±	Equivocal (very faint stain)
1+	Weak (light stain)
2+	Moderate (light-medium stain)
3+	Strong (medium stain)
4+	Intense (dark stain)
Staining Frequency	
SCORE	RESULT
Neg	Negative (no stained cells)
Rare	≤ 5% stained cells of a particular cell type or tissue element
Rare to Occasional	>5-25% stained cells of a particular cell type or tissue element
Occasional	>25-50% stained cells of a particular cell type or tissue element
Occasional to Frequent	>50-75% stained cells of a particular cell type or tissue element
Frequent	>75-100% stained cells of a particular cell type or tissue element

Curve	ka (1/Ms)	kd (1/s)	KD (M)	Rmax (RU)	Conc (M)	tc	Flow (ul/m	kt (RU/Ms)	RI (RU)	Chi² (RU²)	U-value
	9.46E+06	0.002073	2.19E-10	12.16		2.37E+06				0.0125	2
Cycle: 7					2.50E-09		50	8.72E+06	-0.06977		
					5.00E-09				-0.2071		
					1.00E-08				-0.02491		
					2.00E-08				-0.4605		
					4.00E-08				0.08861		

Curve	ka (1/Ms)	SE(ka)	kd (1/s)	SE(kd)	Rmax (RU)	SE(Rmax)	Conc (M)	tc	SE(tc)	f (ul/min)	RI (RU)	SE(RI)
	9.46E+06	7.60E+04	2.07E-03	1.70E-05	12.20	4.60E-03		2.37E+06	2.60E+03			
Cycle: 7							0.00			50.00	-0.10	0.00
							0.00				-0.20	0.00
							0.00				0.00	0.01
							0.00				-0.50	0.01
							0.00				0.10	0.01

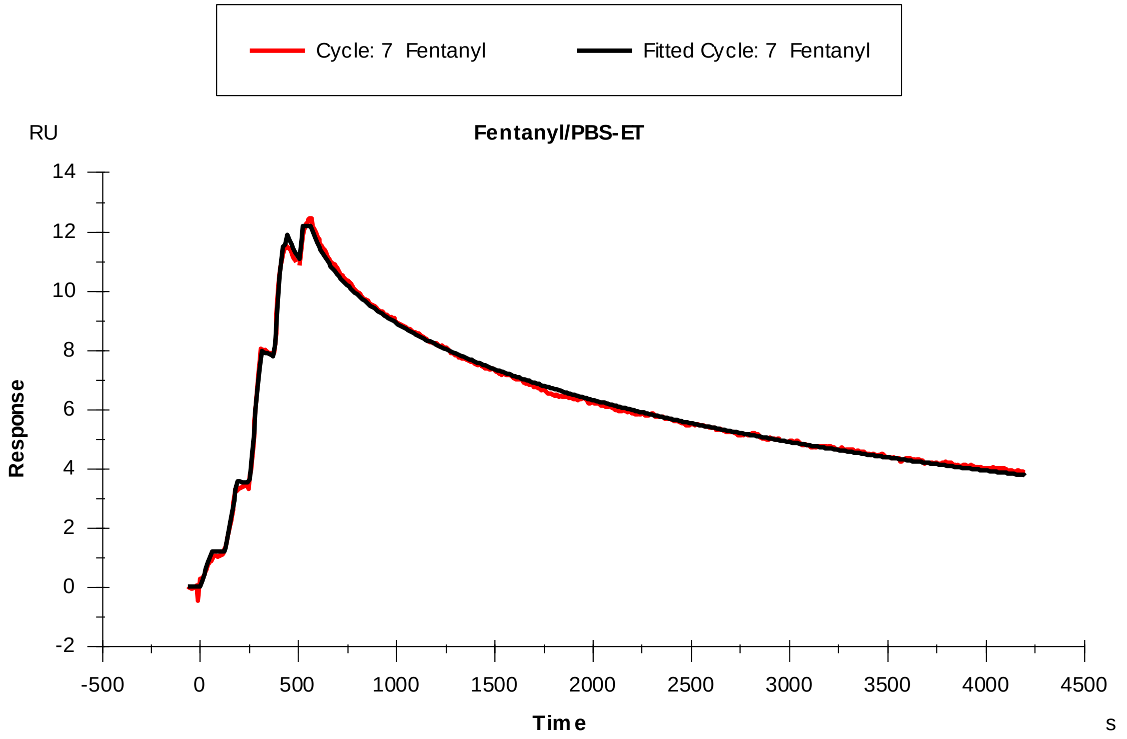

Fig. S1. Representative sensorgram of opioid single-cycle kinetic experiment.

Fentanyl was injected into a CSX-1004 coated sensor at 2.5, 5, 10, 20 and 40 nM, and the data were fit to obtain k_a, k_d and K_D. The experiment was repeated for all other opioids with antibody affinity in the low nanomolar range.

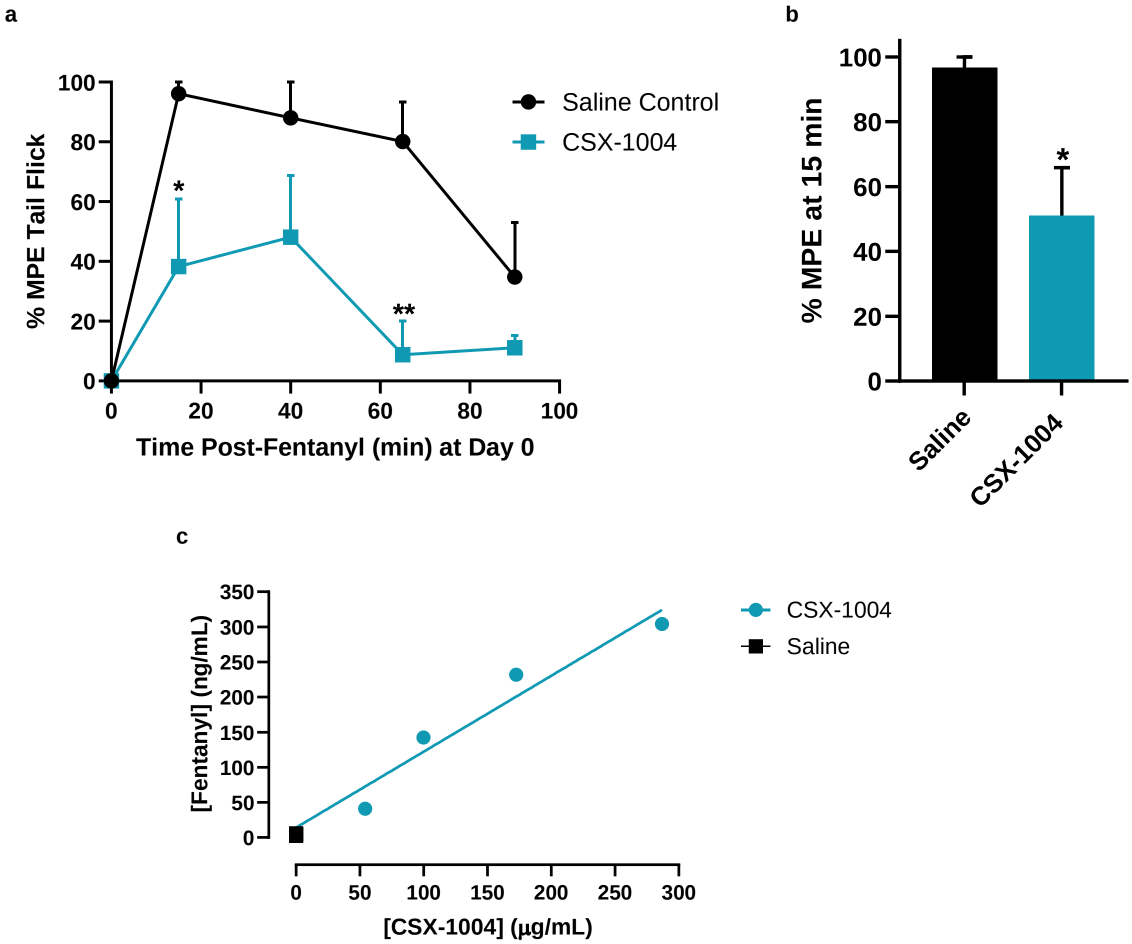

Fig. S2. CSX-1004 prevents fentanyl antinociception in mice.

a Mice were pretreated with 46 mg/kg CSX-1004 IP, and 4 h later antinociception from 0.4 mg/kg IP fentanyl was evaluated. Significant treatment effect in by two-way RM ANOVA [F (1, 6) = 7.491; P = 0.0339]. *P = 0.0213, **P = 0.0031 by Bonferroni's post-hoc test. **b** Antinociception was redetermined 48 h later. Significant treatment effect in n = 4 mice/group at 15-min post 0.4 mg/kg fentanyl by two-tailed t-test; *P = 0.0232. Points and bars represent group means ± SEM. **c** Plot and linear regression of serum antibody concentrations vs. fentanyl concentrations in samples obtained at 20 min following 0.4 mg/kg IP fentanyl (48 h post-CSX-1004). R-squared = 0.9279, Pearson correlation P = 0.0367.

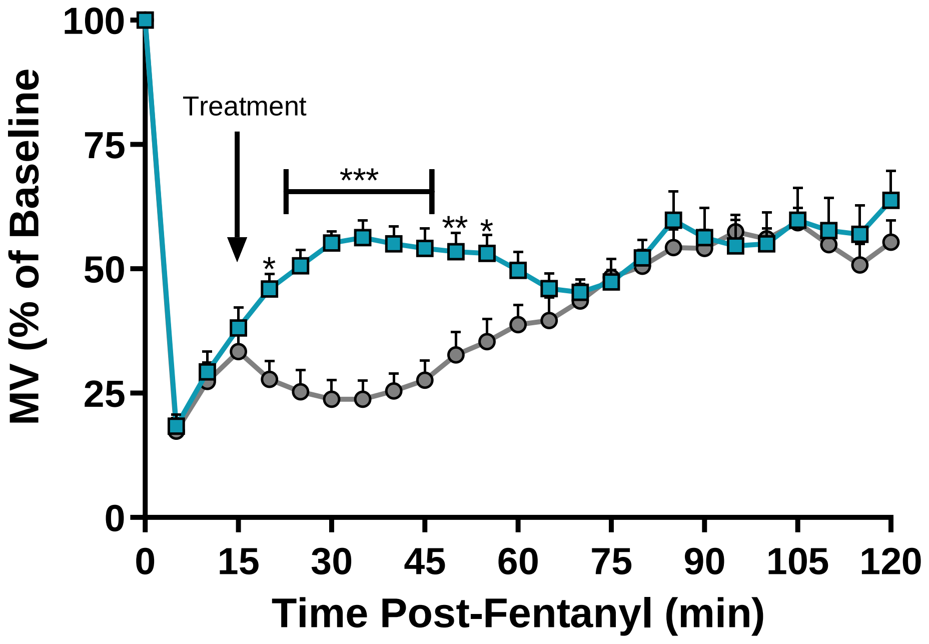

Fig. S3. CSX-1004 rescues fentanyl-induced respiratory depression.

Mice (n=10 per group) received IV fentanyl (0.135 mg/kg) at t = 0 and IV saline or CSX-1004 (60 mg/kg) at t = 15 min. A two-way RM ANOVA showed a significant effect of treatment: $F_{(1, 18)} = 8.053$, $P=0.0109$; Tukey's post-hoc test was used to assess differences between CSX-1004 and saline (*$P<0.05$, **$P<0.01$, ***$P<0.001$).

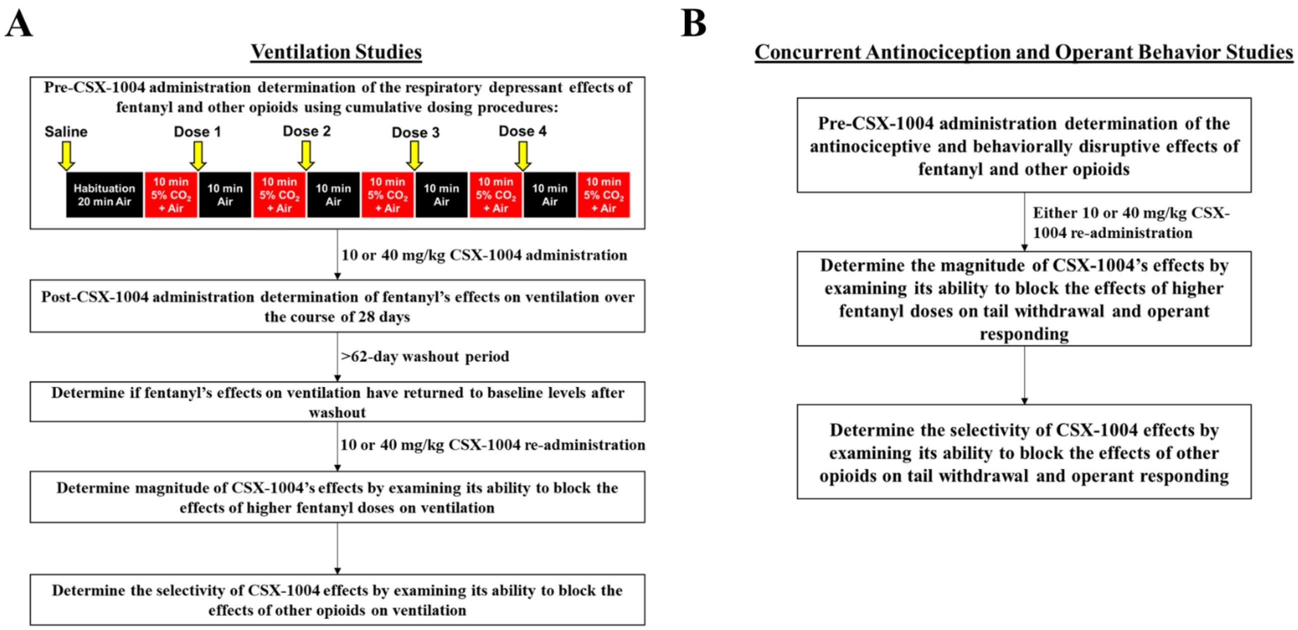

Fig. S4. Flowchart illustration of squirrel monkey experiments.
a Ventilation workflow and **b** concurrent antinociception and behavioral disruption workflow. Effects of cumulative injections of opioids on minute volume in air or air mixed with 5% CO_2 and antinociception/response rates before, during, and after treatment with 10 or 40 mg/kg IV mAb were determined in squirrel monkeys for each endpoint.

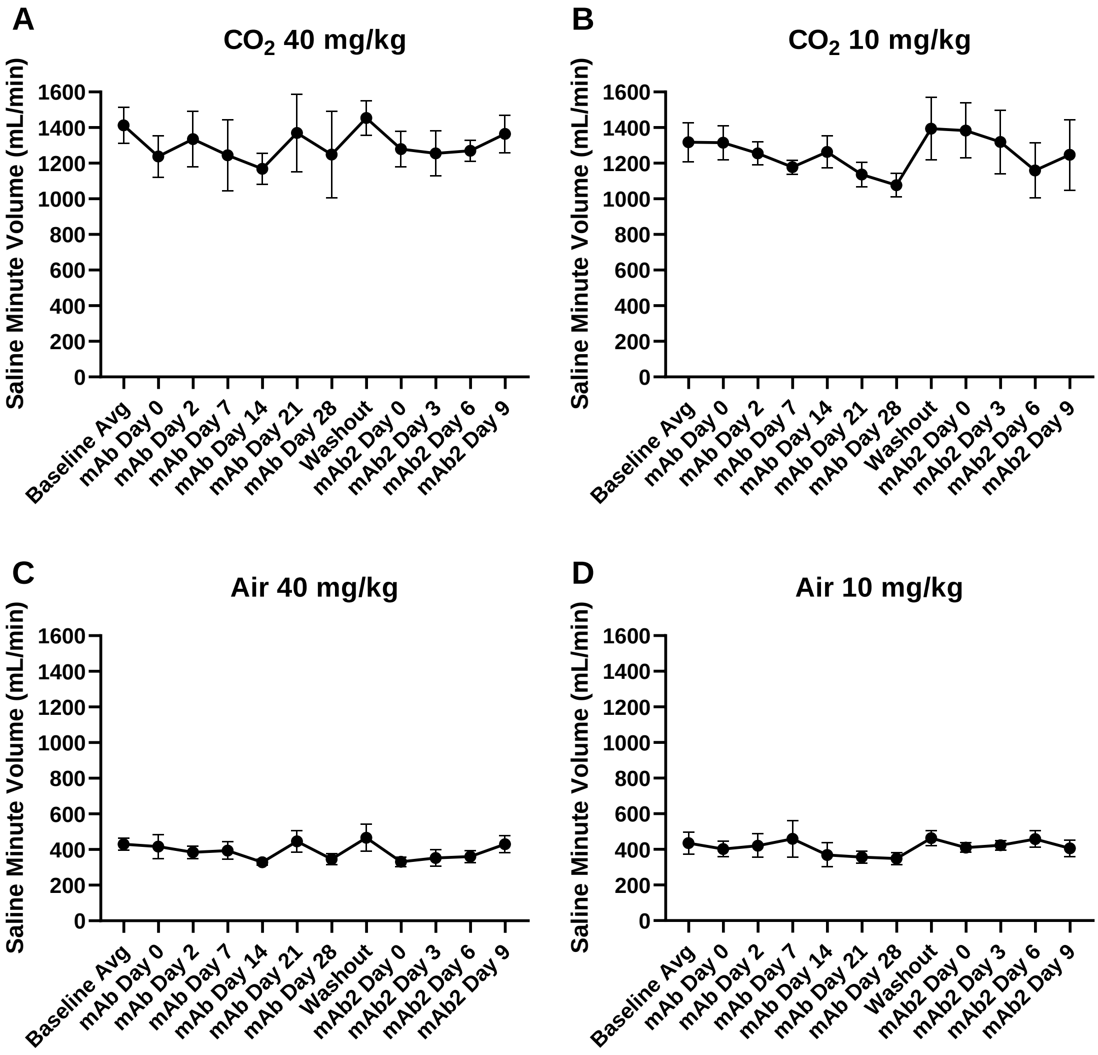

Fig. S5. NHP minute volume in response to saline injections over time.

Minute volumes in NHP from saline injections over the CSX-1004 treatment (10 and 40 mg/kg) time course in air or 5% CO_2 + air atmospheres. The time points are represented relative to the times of first and second CSX-1004 administrations (Day 0) and the washout occurred between 62-128 days. The baseline data point is expressed as the average of 5 measurements. Data are categorized by the indicated atmosphere and CSX-1004 dose. Statistics were assessed by a one-way RM ANOVA for each n = 4 group: **a**, $F (11, 30) = 0.3407$, $p = 0.9688$ **b**, $F (11, 31) = 1.953$, $p = 0.0704$ **c**, $F (11, 30) = 1.049$, $p = 0.4315$ **d**, $F (11, 31) = 0.9239$, $p = 0.5307$. Comparisons vs. baseline were assessed by Dunnett's post-hoc test (no significance noted, $p>0.05$).

	Repeats		
	1	2	3
Sample Name	Response (RU)	Response (RU)	Response (RU)
30 µM Naloxone	83.33	69.07	67.73
30 µM Naltrexone	71.11	66.89	52.76
21 µM Buprenorphine	65.91	62.88	60.49
24 µM Norbuprenorphine	64.04	53.15	57.97
20 µM Oxycodone	60.09	59.65	54.98
20 µM Hydrocodone	60.4	57.84	54.87
20 µM Alfentanil	60.08	57.15	54.95
2 µM Remifentanil	38.67	38.67	38.82
2 µM 6-Acetylmorphine	64.52	61.08	59.22
15.6 nM Fentanyl	4.25	3.25	3.58
15.6 nM Carfentanil	0.46	-7.93	-3.16
No Compound Control	81.4	75.18	74.33
No Compound Control	141.98	135.41	132.47
10 µM Oxymorphone	132.73	133.43	130.44
10 µM Hydromorphone	129.57	130.31	129.18
10 µM Methadone	129.77	129.64	127.04
10 µM Morphine	128.98	129.47	126.22
10 µM U-47700	127.69	128.17	125.52
10 µM Codeine	129.19	127.72	126.75
10 µM Enkephalin	128.82	128.06	110.9
10 µM β-Endorphin	136.63	134.21	136.11
10 µM Dynorphin A	253.61	211.28	187.91
10 nM Fentanyl	-11.41	-1.54	-2.32

Table S1. Raw SPR signals for binding selectivity determination.

Experiments were conducted using a fentanyl-BSA coated sensor chip. Compounds were tested in triplicate at the indicated concentrations in two batches separated by a bold line in the above table.

Sample Time Point (Hours Post Dose)	Group (Sex)	Day 1 CSX-1004 Results			Day 64 CSX-1004 Results		
		Mean Titer (ng/mL)	SD	N	Mean Titer (ng/mL)	SD	N
48	2 (M)	115908	23081.2	3	198644	15395.0	3
	2 (F)	159127	47084.0	3	135789	40001.5	3
	3 (M)	1038739	128651.9	3	1558235	260331.2	3
	3 (F)	895331	242519.4	3	702367	260301.0	3
	4 (M)	3347886	1415747.2	3	4210444	995517.4	3
	4 (F)	4919918	2534041.9	3	4281848	728056.5	3
96	2 (M)	121820	19854.1	3	157858	33015.3	3
	2 (F)	190255	93005.1	3	98604	134435.1	3
	3 (M)	1232645	336008.9	3	793380	419408.6	3
	3 (F)	1170390	586422.4	3	991840	290642.6	3
	4 (M)	4165284	2384642.2	3	3,081,973	741603.7	3
	4 (F)	6504372	1446724.3	3	2697208	110561.9	3
144	2 (M)	113538	55837.3	3	147683	26947.0	3
	2 (F)	115819	16624.9	3	113208	33193.2	3
	3 (M)	1255510	201972.2	3	1024409	79842.8	3
	3 (F)	1548493	383867.4	3	827508	706909.1	3
	4 (M)	2750472	1232163.1	3	2689914	209716.3	3
	4 (F)	4522500	1477512.1	3	2438992	731396.2	3
240	2 (M)	56963	12944.4	3	174572	33363.9	3
	2 (F)	119964	66896.9	3	121094†	-	1
	3 (M)	1170435	732813.0	3	336297	287735.5	3
	3 (F)	2541071	729349.7	3	555,521†	266134.5	2
	4 (M)	3427718	566310.1	3	1450421	531037.3	3
	4 (F)	3002192	103619.0	3	2196074	790700.3	3
288	2 (M)	40765	3636.4	3	95389	27936.2	3
	2 (F)	46576	4573.6	3	54012	17539.9	3
	3 (M)	641049	150499.4	3	640799	51666.9	3
	3 (F)	763553	281775.2	3	385057	13942.0	3
	4 (M)	2216995	1090837.6	3	1764946	175390.6	3
	4 (F)	2917701	166221.4	3	2256209	280646.7	3
408	2 (M)	37732	4893.7	3	63,714	6968.5	3
	2 (F)	96936	35928.2	3	42,959†	-	1
	3 (M)	236709	190633.2	3	318,755	30703.3	3
	3 (F)	538736	123570.2	3	416,150	217976.0	3
	4 (M)	1033830	530007.7	3	1,099,438	570936.8	3
	4 (F)	2260374	677142.8	3	1,267,271	225255.1	3
504	2 (M)	43453	4840.5	3	51,631	8798.5	3
	2 (F)	54795	18075.4	3	26,042	7035.7	3
	3 (M)	246864	173203.3	3	551,025	63326.7	3
	3 (F)	250312	211146.9	3	260,843	6753.6	3
	4 (M)	734591	272487.8	3	1,248,200	162088.2	3
	4 (F)	878570	309190.2	3	953,320	148690.3	3

Table S2. Summary of Serum CSX-1004 Levels

Rats (n=6/sex/group) were administered CSX-1004 at 0, 10, 100 and 400 mg/kg IV on days 0, 21, 42 and 63 and blood sampling was performed for n=3/sex/group per timepoint, and samples were analyzed by ELISA. “SD” = Standard Deviation; “N” = Number of Animals; “-“ = Not calculable; † = Results less than the smallest standard value (<SSV) not included in mean titer calculation.

Endpoints	Dose	10 mg/kg		40 mg/kg	
Respiration	Atmosphere	Air	5% CO2	Air	5% CO2
	Pre-mAb ED_{50} ± SEM; mg/kg	0.019 ± 0.003	0.015 ± 0.001	0.026 ± 0.004	0.012 ± 0.003
	mAb ED_{50} ± SEM; mg/kg	0.101 ± 0.002	0.066 ± 0.012	0.310 ± 0.093	0.162 ± 0.053
	Fold Shift ± SEM	5.33 ± 0.75	4.52 ± 0.89	11.9 ± 4.0	13.7 ± 5.6
	Washout ED_{50} ± SEM; mg/kg	0.019 ± 0.009	0.014 ± 0.002	0.024 ± 0.009	0.011 ± 0.002
Tail Withdrawal	Pre-mAb ED_{50} ± SEM; mg/kg	0.0056 ± 0.0054 [0.0038 ± 0.0004]			
	mAb ED_{50} ± SEM; mg/kg	0.062 ± 0.091 [0.122 ± 0.095]		0.075 ± 0.127 [0.067 ± 0.010]	
	Fold Shift ± SEM	11.0 ± 19.3 [32.1 ± 25.2]		13.3 ± 25.9 [17.6 ± 3.2]	
	Washout ED_{50} ± SEM; mg/kg	0.0057 ± 0.0054 [0.0038 ± 0.0007]			
Operant Responding	Pre-mAb ED_{50} ± SEM; mg/kg	0.00476 ± 0.00044			
	mAb ED_{50} ± SEM; mg/kg	0.023 ± 0.010		0.065 ± 0.010	
	Fold Shift ± SEM	4.8 ± 2.2		15.8 ± 3.3	
	Washout ED_{50} ± SEM; mg/kg	0.0042 ± 0.0006			

Table S3. Fentanyl ED_{50} values and corresponding fold shifts.

ED_{50} values ± SD were calculated using linear regression analysis with no data exclusions. Since %CV values exceeded 200 for tail withdrawal, a non-linear curve was fit yielding ED_{50}s with much lower %CV (shown in brackets).

Endpoint	Respiration			Tail Withdrawal			Response Rate		
	Pre-mAb	10 mg/kg	40 mg/kg	Pre-mAb	10 mg/kg	40 mg/kg	Pre-mAb	10 mg/kg	40 mg/kg
Alfentanil ED_{50} ± SEM (mg/kg)	0.058 ± 0.007	0.061 ± 0.013	0.059 ± 0.011	0.031 ± 0.028	0.042 ± 0.099	0.034 ± 0.016	0.036 ± 0.006	0.032 ± 0.009	0.027 ± 0.004
Alfentanil Fold Shift ± SEM		1.047 ± 0.258	1.019 ± 0.225		1.335 ± 3.358	1.074 ± 1.075		0.885 ± 0.296	0.766 ± 0.177
Morphine ED_{50} ± SEM (mg/kg)	1.146 ± 0.104	1.219 ± 0.123	1.007 ± 0.086	0.923 ± 0.695	1.220 ± 3.761	0.982 ± 10.829	0.997 ± 0.162	0.963 ± 0.170	0.863 ± 0.137
Morphine Fold Shift ± SEM		1.064 ± 0.145	0.879 ± 0.110		1.322 ± 4.194	1.064 ± 11.758		0.966 ± 0.232	0.865 ± 0.197
Oxycodone ED_{50} ± SEM (mg/kg)	0.475 ± 0.069	0.609 ± 0.142	0.475 ± 0.047	0.317 ± 0.719	0.322 ± 3.296	0.389 ± 1.033	0.340 ± 0.055	0.284 ± 0.031	0.363 ± 0.059
Oxycodone Fold Shift ± SEM		1.283 ± 0.353	0.999 ± 0.176		1.016 ± 10.637	1.227 ± 4.281		0.836 ± 0.162	1.068 ± 0.243

Table S4. ED_{50} values and corresponding fold shifts for non-fentanyl opioids.

ED_{50} values ± SD were calculated using a linear regression analysis with no data exclusions.

Supplementary References

1. K. M. Fung, A. Messing, V. M. Lee, J. Q. Trojanowski, A novel modification of the avidin-biotin complex method for immunohistochemical studies of transgenic mice with murine monoclonal antibodies. *J Histochem Cytochem* **40**, 1319-1328 (1992).
2. B. P. Hierck, L. V. Iperen, A. C. Gittenberger-De Groot, R. E. Poelmann, Modified indirect immunodetection allows study of murine tissue with mouse monoclonal antibodies. *J Histochem Cytochem* **42**, 1499-1502 (1994).
3. J. R. Tuson, E. W. Pascoe, D. A. Jacob, A novel immunohistochemical technique for demonstration of specific binding of human monoclonal antibodies to human cryostat tissue sections. *J Histochem Cytochem* **38**, 923-926 (1990).
