# Supplementary material for: Investigation of monoclonal antibody CSX-1004 for fentanyl overdose
Source: Nat Commun. 2023 Dec 5;14:7700. doi: 10.1038/s41467-023-43126-0 (PMC10698161; doi:10.1038/s41467-023-43126-0)
Supplement: Supplementary file 2 — Description of Additional Supplementary Files [file 41467_2023_43126_MOESM2_ESM.pdf]

Description of Additional Supplementary Files

Title: Supplementary Data 1

Description: Anti-drug antibody assay results and tissue cross-reactivity assay results
