# Supplementary material for: Investigation of monoclonal antibody CSX-1004 for fentanyl overdose
Source: Nat Commun. 2023 Dec 5;14:7700. doi: 10.1038/s41467-023-43126-0 (PMC10698161; doi:10.1038/s41467-023-43126-0)
Supplement: Supplementary file 4 — Reporting Summary [file 41467_2023_43126_MOESM4_ESM.pdf]

nature portfolio

Corresponding author(s): Paul T. Bremer
Cessation Therapeutics, Inc.
San Diego, CA
Email: pbremer@cessationtx.com

Last updated by author(s): 10/26/2023

Reporting Summary

Nature Portfolio wishes to improve the reproducibility of the work that we publish. This form provides structure for consistency and transparency in reporting. For further information on Nature Portfolio policies, see our Editorial Policies and the Editorial Policy Checklist.

Statistics

For all statistical analyses, confirm that the following items are present in the figure legend, table legend, main text, or Methods section.

n/a	Confirmed	
☐	☒	The exact sample size (*n*) for each experimental group/condition, given as a discrete number and unit of measurement
☐	☒	A statement on whether measurements were taken from distinct samples or whether the same sample was measured repeatedly
☐	☒	The statistical test(s) used AND whether they are one- or two-sided<br>*Only common tests should be described solely by name; describe more complex techniques in the Methods section.*
☒	☐	A description of all covariates tested
☐	☒	A description of any assumptions or corrections, such as tests of normality and adjustment for multiple comparisons
☐	☒	A full description of the statistical parameters including central tendency (e.g. means) or other basic estimates (e.g. regression coefficient) AND variation (e.g. standard deviation) or associated estimates of uncertainty (e.g. confidence intervals)
☐	☒	For null hypothesis testing, the test statistic (e.g. *F*, *t*, *r*) with confidence intervals, effect sizes, degrees of freedom and *P* value noted<br>*Give P values as exact values whenever suitable.*
☒	☐	For Bayesian analysis, information on the choice of priors and Markov chain Monte Carlo settings
☒	☐	For hierarchical and complex designs, identification of the appropriate level for tests and full reporting of outcomes
☒	☐	Estimates of effect sizes (e.g. Cohen's *d*, Pearson's *r*), indicating how they were calculated

Our web collection on statistics for biologists contains articles on many of the points above.

Software and code

Policy information about availability of computer code

Data collection	Plethysmography SCIREQ IOX2 software by EMKA, Operant Responding Med-PC ver. 4 by Med Associates Inc., SPR (Biacore S200 ver. 1.1 software), ELISA (SoftMax Pro ver. 7), LCMS (Waters MassLynx ver. 4.1)
Data analysis	PK analysis was performed using PKAnalix ver 2021R2 and Phoenix WinNonLin ver. 8.3. All statistical data analyses were performed using GraphPad Prism 8.

For manuscripts utilizing custom algorithms or software that are central to the research but not yet described in published literature, software must be made available to editors and reviewers. We strongly encourage code deposition in a community repository (e.g. GitHub). See the Nature Portfolio guidelines for submitting code & software for further information.

Data

Policy information about availability of data

All manuscripts must include a data availability statement. This statement should provide the following information, where applicable:

- Accession codes, unique identifiers, or web links for publicly available datasets
- A description of any restrictions on data availability
- For clinical datasets or third party data, please ensure that the statement adheres to our policy

All data supporting the main conclusions of the paper are present in the main text and supporting info. Additional information is available upon request from the authors. The raw data generated in this study have been deposited in the Figshare database.

Research involving human participants, their data, or biological material

Policy information about studies with human participants or human data. See also policy information about sex, gender (identity/presentation), and sexual orientation and race, ethnicity and racism.

Reporting on sex and gender	Not Applicable
Reporting on race, ethnicity, or other socially relevant groupings	Not Applicable
Population characteristics	Not Applicable
Recruitment	Not Applicable
Ethics oversight	The GLP human tissue cross-reactivity study was approved and conducted by Charles River Laboratories in accordance with U.S. Department of Health and Human Services (HHS) and Food and Drug Administration (FDA).

Note that full information on the approval of the study protocol must also be provided in the manuscript.

Field-specific reporting

Please select the one below that is the best fit for your research. If you are not sure, read the appropriate sections before making your selection.

- [x] Life sciences
- [ ] Behavioural & social sciences
- [ ] Ecological, evolutionary & environmental sciences

For a reference copy of the document with all sections, see nature.com/documents/nr-reporting-summary-flat.pdf

Life sciences study design

All studies must disclose on these points even when the disclosure is negative.

Sample size	Sample values were not pre-calculated. n=6-12 in mouse studies (n=4 for supplementary study) and n=12-40 in rat studies. A sample size of at least 4 was used for NHP studies. Sample size, sex, species and strain are noted for each experiment. Historical data has indicated that pharmacokinetic and behavioral data in, respectively, rodents and monkeys with the sample sizes used in this study provides a power level of 80% for these studies.
Data exclusions	No data were excluded from the analysis.
Replication	To ensure reproducibility, baseline respiration data were collected at 12 different time points in NHP subjects (Fig S5) and full fentanyl dose response curves were redetermined after washout periods. All repeat measurements demonstrated data consistency.
Randomization	Pseudorandomization was performed across subjects accounting for sex, age and bodyweight, except rats which were assigned to groups via a computerized, randomization procedure.
Blinding	The present studies were conducted unblinded to treatment condition to facilitate efficient response to untoward effects of high doses of administered opioid drugs. In addition, all experimental variables were controlled by automated instrumentation/software, and therefore blinding to treatment conditions were not necessary.

Reporting for specific materials, systems and methods

We require information from authors about some types of materials, experimental systems and methods used in many studies. Here, indicate whether each material, system or method listed is relevant to your study. If you are not sure if a list item applies to your research, read the appropriate section before selecting a response.

Materials & experimental systems

n/a	Involved in the study	
[ ]	[x]	Antibodies
[x]	[ ]	Eukaryotic cell lines
[x]	[ ]	Palaeontology and archaeology
[ ]	[x]	Animals and other organisms
[x]	[ ]	Clinical data
[x]	[ ]	Dual use research of concern
[x]	[ ]	Plants

Methods

n/a	Involved in the study	
[x]	[ ]	ChIP-seq
[x]	[ ]	Flow cytometry
[x]	[ ]	MRI-based neuroimaging

Antibodies

Antibodies used	CSX-1004 was manufactured by KBI Biopharma (lots 210618-0040-BDS and 211116-0101-BDS) as a 100 mg/mL solution, and testing results of these lots met or exceeded minimum acceptance criteria for appearance (clear, slightly yellow, free of visible particles), pH, purity (SEC HPLC main peak: 98.3-98.8%, reduced CE-SDS: 97.8%), potency (100% fentanyl-BSA binding vs. reference material) and strength. Other antibodies include 1:10,000 goat anti-human IgG HRP (1 mg/mL, Southern Biotech #204005, Birmingham, AL), 1:10,000 goat anti-human IgG Fc Specific-HRP (1 mg/mL Sigma A-0170), and 5-15 ug/mL human IgG1 kappa Abcam (Catalog No. ab206198).
Validation	Testing to establish CSX-1004 quality, purity, potency, strength and safety were performed as per ICH guidelines mandated by FDA.

Animals and other research organisms

Policy information about studies involving animals; ARRIVE guidelines recommended for reporting animal research, and Sex and Gender in Research

Laboratory animals	Animals used were Swiss Webster mice (6-8 weeks old), Sprague-Dawley rats (8 weeks old) and 5 male/3 female NHPs (Saimiri sciureus, 8-18 years old).
Wild animals	The study did not involve wild animals.
Reporting on sex	Experiments conducted in mice were all female. Rats were equal numbers of male and female. A group of eight adult squirrel monkeys (5 Males/3 Females) were used in this study. The uneven sample size of males and females is because NHPs being a valuable resource and there are limitations in availability of these subjects. Only one sex was used in mouse studies because they are considered proof of concept studies for which results are not considered to be affected by sex. Studies in rats did not detect any significant impact of sex on CSX-1004 toxicokinetic endpoints.
Field-collected samples	The study did not involve samples collected from the field.
Ethics oversight	All animal procedures were approved by the Institutional Animal Care and Use Committees (IACUC) at McLean Hospital, Scripps Research Institute and Illinois Institute of Technology Research Institute for monkeys, mice and rats, respectively. Monkeys were housed in a climate-controlled vivarium with a 12-h light/dark cycle (lights on at 07:00AM – 7:00PM) in the McLean Hospital Animal Care Facility. Mice were housed in a facility kept at 22-26°C and 60–70% humidity with a 12/12 h light–dark cycle and were provided access ad libitum access to food and water. Rats were housed in a facility kept at 20-26°C and 30-70% humidity with a 12/12 h light-dark cycle and were given a specified amount of food daily (Envigo 2014C) with ad libitum access to water, both of which were routinely tested for pathogens. Mice were housed in a climate controlled vivarium [(temperature = 72°F, range: ~64 – 84°F); relative humidity = 50%, range: 40 – 70%)] on a 12 h light/dark cycle (lights on at 7 AM – 7PM) with ad libitum access to water and food (Inotiv #2018).

Note that full information on the approval of the study protocol must also be provided in the manuscript.
