# Supplementary material for: Investigation of monoclonal antibody CSX-1004 for fentanyl overdose
Source: Nat Commun. 2023 Dec 5;14:7700. doi: 10.1038/s41467-023-43126-0 (PMC10698161; doi:10.1038/s41467-023-43126-0)
Supplement: Supplementary file 5 — Peer Review [file 41467_2023_43126_MOESM5_ESM.pdf]

nature portfolio

Peer Review File

Investigation of a Novel Monoclonal Antibody CSX-1004 for Fentanyl Overdose

Open Access This file is licensed under a Creative Commons Attribution 4.0 International License, which permits use, sharing, adaptation, distribution and reproduction in any medium or format, as long as you give appropriate credit to the original author(s) and the source, provide a link to the Creative Commons license, and indicate if changes were made. In the cases where the authors are anonymous, such as is the case for the reports of anonymous peer reviewers, author attribution should be to 'Anonymous Referee' followed by a clear attribution to the source work. The images or other third party material in this file are included in the article's Creative Commons license, unless indicated otherwise in a credit line to the material. If material is not included in the article's Creative Commons license and your intended use is not permitted by statutory regulation or exceeds the permitted use, you will need to obtain permission directly from the copyright holder. To view a copy of this license, visit http://creativecommons.org/licenses/by/4.0/.

REVIEWER COMMENTS

Reviewer #1 (Remarks to the Author):

Review of NCOMMS-23-33330

Summary. This study reports development and IND enabling studies of a monoclonal antibody (mAB) for rescue of fentanyl overdose. Results reported include the GLP toxicology and safety profiles of this product.

Main article.

1) Abstract mentioned respiratory depression in NHP, but supplementals also mention operant studies in NHP, please clarify or revise text for consistency.
2) Page 2, line 45 paragraph: please add nalmefene in the introduction alongside naloxone.
3) Ln64 "currently no FDA-approved products" please revise. Some products like buprenorphine can prevent to some extent overdoses from opioids
4) References 19-22: are all published fentanyl mAb studies correctly cited? Please verify.
5) LN70: CSX-1004 is a fully human… please provide a bit more details about this mAb. Was this previously disclosed in Smith et al. from the Janda group?
6) LN115: were the doses (10, 100, 400 mg/kg mAb) chosen based upon future Phase I ascending dose studies, can the authors elaborate on that?
7) LN142: "0.001 to 0.018 mg/kg" are these relevant doses in opioid tolerant individuals? Or do you foresee these doses modeling an opioid naïve human subject?
8) LN255: can the authors discuss the economical viability of mAbs which could be more expensive than vaccines?

Supplemental Materials

1) In regard to mouse studies: why the mAb and fentanyl were administered IP instead of a more relevant route of administration such as SC or IV? But I then acknowledge that Figure S3 has data obtained with IV challenges of fentanyl and antibody in rescue.
2) In regards to "Blood samples for determination of anti-CSX-1004 antibodies (via anti drug antibody ELISA)", and the related description of ADA detection on page 10. The coating antigen Fentanyl-BSA + CSX-1004 as detection setup would be very effective in detection of

anti-CSX-1004 antibodies directed against the Fc portion of the CSX-1004, but less so for detection of Fv portion of the CSX-1004. Please discuss this possibility of underestimating the detection of ADA.

3) Table S4. Did the studies yield sufficient timepoints to measure ED50 values ± SD and the shift pre- post- administration of the antibody?

4) Table S5: pre-mAb vs post- ED50 ± SEM (mg/kg) shows that mAb doesn't bind morphine, oxycodone, and alfentanil. Is there an explanation that alfentanil was not bound?

Questions (as asked by the journal)

What are the noteworthy results? advancement of a novel mAb towards IND stage

Will the work be of significance to the field and related fields?

yes, as it will open the path to new biologics for OUD and overdose to transition to clinical trials

How does it compare to the established literature? If the work is not original, please provide relevant references.

Other studies have published mAbs against opioids, but this study adds new pharmacology and behavioral aspects in NHP

Does the work support the conclusions and claims, or is additional evidence needed?

yes, it supports the conclusions and claims

Are there any flaws in the data analysis, interpretation and conclusions? Do these prohibit publication or require revision?

Minor revisions are suggested above

Is the methodology sound? Does the work meet the expected standards in your field?

yes

Is there enough detail provided in the methods for the work to be reproduced?

yes

Reviewer #2 (Remarks to the Author):

This study investigated ability of a CSX-1004, a fentanyl targeting monoclonal antibody, to reverse and prevent clinically relevant effects of opioids, particularly antinociceptive and respiratory-depressant effects, as well as characterize pharmacokinetic and toxicological profile. Using several relevant procedures in different species, including nonhuman primates (squirrel monkeys), these studies showed that CSX-1004 selectively blocked effects of fentanyl and carfentanil, without altering effects of non-fentanyl drugs such as oxycodone and morphine, and did so in time- and dose-related manner. Overall, the set of studies are rigorously conducted using relevant procedures with cross-species and within-subject generalization of results. Different behavioral endpoints and dose-response functions were employed to more fully characterize interactions between the antibody and opioids. Importantly, studies demonstrate effectiveness in primates increasing confidence in successful translation of these results to the clinic. This paper will make an important contribution to the literature and should be of interest to a broad readership. Below are a few comments for consideration in revision.

1. "…to rescue mice from fentanyl-induced antinociception and respiratory depression." Reconsider the use of rescue here, especially as it relates to antinociceptive effects. Perhaps "reverse"?

2. "A rapid reversal…within 20 minutes…" Not sure this should be characterized as "rapid", given the need for quick reversal of fentanyl effects noted in the Introduction.

3. "While naloxone was initially more effective at reversing respiratory-depression, CSX-1004 markedly surpassed the magnitude of naloxone…" Might not a larger dose of naloxone

be expected to produce a greater magnitude of effect (i.e., full reversal)? The comparison of magnitude of effect in the absence of dose-effect relationships, including an asymptote at the maximum effect, is tenuous.

4. Figures 2a and 2b: recommend having ticks on the actual time points, rather than forcing the reader to interpolate.

5. Results note differences in the magnitude of antagonism. Were shifts compared statistically against each other, or are these conclusions based on nominal differences in the mean for the group?

<u>Response To Reviewers:</u>

<u>**Reviewer #1:**</u> **Summary.**
This study reports development and IND enabling studies of a monoclonal antibody (mAb) for rescue of fentanyl overdose. Results reported include the GLP toxicology and safety profiles of this product.

Main article.
1) Abstract mentioned respiratory depression in NHP, but supplementals also mention operant studies in NHP, please clarify or revise text for consistency.

We thank the reviewer for pointing this out. Text in the abstract has been revised to include operant studies.

"Furthermore, treatment with CSX-1004 dose-dependently produced up to a 15-fold rightward shift in NHP respiration, antinociception and operant responding assays…"

2) Page 2, line 45 paragraph: please add nalmefene in the introduction alongside naloxone.

Nalmefene has been added alongside naloxone in the revised manuscript.

"…although a much longer acting naloxone-like medication known as nalmefene has been FDA-approved this year to combat F/FA overdose."

3) Ln64 "currently no FDA-approved products" please revise. Some products like buprenorphine can prevent to some extent overdoses from opioids.

While it is true that buprenorphine can provide some overdose protection, it has not been FDA-approved for this specific indication. We have added some additional language to clarify this.

"…although buprenorphine has been shown to mitigate fentanyl-induced respiratory depression.[32]"

4) References 19-22: are all published fentanyl mAb studies correctly cited? Please verify.

Thank you for bringing this up. There have been some recent publications related to fentanyl mAbs that we have now added to the citations. Regarding the vaccines, we were not comprehensive in citing all of them since this paper is about a mAb. Please let us know if we have missed any.

23. L. M. Eubanks, T. Pholcharee, D. Oyen, Y. Natori, B. Zhou, I. A. Wilson, K. D. Janda, An Engineered Human-Antibody Fragment with Fentanyl Pan-Specificity That Reverses Carfentanil-Induced Respiratory Depression. ACS Chem Neurosci 14, 2849-2856 (2023).
24. M. Lin, L. M. Eubanks, N. M. Karadkhelkar, S. Blake, K. D. Janda, Catalytic Antibody Blunts Carfentanil-Induced Respiratory Depression. ACS Pharmacol Transl Sci 6, 802-811 (2023).

25. J. V. Rodarte, C. Baehr, D. Hicks, T. L. Liban, C. Weidle, P. B. Rupert, R. Jahan, A. Wall, A. T. McGuire, R. K. Strong, S. Runyon, M. Pravetoni, M. Pancera, Structures of drug-specific monoclonal antibodies bound to opioids and nicotine reveal a common mode of binding. Structure 31, 20-32 e25 (2023).
26. G. Triller, E. P. Vlachou, H. Hashemi, M. van Straaten, J. P. Zeelen, Y. Kelemen, C. Baehr, C. L. Marker, S. Ruf, A. Svirina, M. Chandra, K. Urban, A. Gkeka, S. Kruse, A. Baumann, A. K. Miller, M. Bartel, M. Pravetoni, C. E. Stebbins, F. N. Papavasiliou, J. P. Verdi, A trypanosome-derived immunotherapeutics platform elicits potent high-affinity antibodies, negating the effects of the synthetic opioid fentanyl. Cell Reports 42, 112049 (2023).
27. C. A. Baehr, M. M. Wu, S. G. Pandit, J. Arias-Umana, D. AuCoin, M. Pravetoni, Pharmacological Profiling of Antifentanyl Monoclonal Antibodies in Combination with Naloxone in Pre- and Postexposure Models of Fentanyl Toxicity. J Pharmacol Exp Ther 381, 129-136 (2022).
28. E. A. Townsend, P. T. Bremer, N. T. Jacob, S. S. Negus, K. D. Janda, M. L. Banks, A synthetic opioid vaccine attenuates fentanyl-vs-food choice in male and female rhesus monkeys. Drug Alcohol Depend 218, 108348 (2021).
29. R. C. Barrientos, E. W. Bow, C. Whalen, O. B. Torres, A. Sulima, Z. Beck, A. E. Jacobson, K. C. Rice, G. R. Matyas, Novel Vaccine That Blunts Fentanyl Effects and Sequesters Ultrapotent Fentanyl Analogues. Mol Pharm 17, 3447-3460 (2020).
30. M. D. Raleigh, F. Baruffaldi, S. J. Peterson, M. Le Naour, T. M. Harmon, J. R. Vigliaturo, P. R. Pentel, M. Pravetoni, A Fentanyl Vaccine Alters Fentanyl Distribution and Protects against Fentanyl-Induced Effects in Mice and Rats. J Pharmacol Exp Ther 368, 282-291 (2019).
31. P. T. Bremer, A. Kimishima, J. E. Schlosburg, B. Zhou, K. C. Collins, K. D. Janda, Combatting Synthetic Designer Opioids: A Conjugate Vaccine Ablates Lethal Doses of Fentanyl Class Drugs. Angewandte Chemie-International Edition 55, 3772-3775 (2016).

5) LN70: CSX-1004 is a fully human… please provide a bit more details about this mAb. Was this previously disclosed in Smith et al. from the Janda group?

CSX-1004 is a completely different mAb than the mouse-derived antibody (6A4) disclosed in the Janda paper. We have noted that CSX-1004 is a fully human antibody discovered through the use of transgenic OmniRats® that is suitable for clinical use. The 6A4 antibody served as a proof of concept, which would require significant engineering work (humanization) to become suitable for human use. We cited the method used to discover the antibody; however, details beyond that we view as out of scope since this manuscript details pharmacological profiling, safety testing and in vivo efficacy to show its translational potential to be an anti-fentanyl overdose prevention medication.

"…using a previously reported method[23]"

6) LN115: were the doses (10, 100, 400 mg/kg mAb) chosen based upon future Phase I ascending dose studies, can the authors elaborate on that?

Yes, the target doses for our Phase 1 study are 1-40 mg/kg; thus, the toxicology study involving 10-400 mg/kg doses give the appropriate 10-fold safety margin to support a range of starting doses for clinical testing.

For the proposed Phase 1a study, the proposed initial starting clinical dose was determined based on the approach outlined in FDA's 2005 guideline entitled "Estimating the Maximum Safe Starting Dose in Initial Clinical Trials for Therapeutics in Adult Healthy Volunteers." According to this document, for proteins with a molecular weight >100 kDa and that are administered intravascularly, the starting dose should be normalized to mg/kg (not using a human equivalent dose conversion). CSX-1004 fulfills these criteria with a molecular weight of 147 kDa and administration via IV infusion. Applying a 10-fold safety margin to the NOAEL dose (400 mg/kg) in the pivotal, rat GLP toxicology study would support a safe human starting dose of 40 mg/kg. However, out of an abundance of caution as this is a first-in-human study of a biologic agent, the initial dose in the first-in-human study is 1.0 mg/kg, with plans to escalate to a maximum dose of 40 mg/kg in successive cohorts of subjects.

"…providing support for initial human doses up to 40 mg/kg."

7) LN142: "0.001 to 0.018 mg/kg" are these relevant doses in opioid tolerant individuals? Or do you foresee these doses modeling an opioid naïve human subject?

The reviewer raises an interesting point. According to Algera et al. 2021 Clin Pharm & Therapeutics, chronic opioid users showed a 4.3-fold tolerance to fentanyl-induced respiratory depression, but also a risk for potentially fatal apnea at all fentanyl doses tested. This suggests that opioid-tolerant individuals are still at risk for fentanyl lethality at doses administered by opioid-naïve individuals. While the NHPs used in experiments were not considered opioid-tolerant, the very large shifts in the OIRD dose-effect curves >5-15 fold are anticipated to be clinically relevant in offering significant protection to both opioid naive and opioid-tolerant individuals.

Experiments examining rightward shifts in the dose-response curve for fentanyl-induced respiratory depression (Fig. 4) are even more relevant for opioid-tolerant subjects. In these experiments, the highest dose tested, 0.18 mg/kg, is equivalent to **14.4 mg** of fentanyl in an 80 kg human. Such a dose is likely to confer a risk of overdose death in both opioid-naïve and opioid-tolerant individuals. For example, in an analysis of 541 individuals who died of a fentanyl overdose in New Hampshire, the mean plasma fentanyl concentration was 9.96 ng/mL (NDEWS 2017). This concentration corresponds to an intravenous dose of fentanyl of **1.63 mg** (0.02 mg/kg) (Mann et al 2022). Importantly, the New Hampshire study population was comprised of inidividuals with a history of opioid use, including opioid use disorder (63.4%), previous overdose (12.7%), and prescribed on opioid during the previous 12 months (14.8%). Among those with opioid use disorder, 89.7% were reported as injection drug users. Accordingly, the highest dose tested in nonhuman primates was approximately 9-fold higher (14.4 mg/1.63 mg = 8.83) than doses that produce lethality in opioid-tolerant humans. While acknowledging that opioid tolerance can be profound and widely discrepant across individuals, it appears that the doses tested in nonhuman primates are relevant for many opioid-tolerant individuals.

We have added some additional text to clarify this.

"We consider the 5-15 fold fentanyl potency shifts in the NHP model requiring up to 0.18 mg/kg fentanyl (equal to 14.4 mg in a 80 kg adult) to be clinically relevant for overdose prevention regardless of opioid tolerance. While chronic opioid users exhibit a 4.3-fold reduced sensitivity to fentanyl-induced respiratory depression, the doses tested in NHPs are considered lethal regardless of opioid history.[9, 55]"

Furthermore, this edited section provides relevant information:

"Compared to monkeys, humans are somewhat more sensitive to opioids (the monkey OIRD ED_{50} of ~0.02 mg/kg fentanyl is similar to the fentanyl LD_{50} in humans)[35, 48, 49]; thus, a larger CSX-1004-mediated fentanyl potency shift in humans could be obtained because of the higher potency of fentanyl in humans coupled with the greater predicted exposure (AUC) of the mAb in humans vs. monkeys."

8) LN255: can the authors discuss the economical viability of mAbs which could be more expensive than vaccines?

We don't believe that economic considerations are particularly relevant to our manuscript because its primary purpose is to investigate the efficacy and safety of our mAb for combatting fentanyl's effects. We have yet to conduct clinical trials with CSX-1004, and thus any price considerations are premature. However, we have added to the following text to address the reviewer's query:

"A potential limitation to anti-opioid mAbs vs. small-molecule medications or vaccines is the higher manufacturing and administration costs,[53] but the cost of mAbs varies greatly across therapeutic category. Anti-opioid mAbs could reasonably be priced on par with currently marketed, once monthly treatments for OUD. Finally, medications for OUD enjoy broad coverage by payers including Medicare and Medicaid.[54, 55]"

Supplemental Materials

1) In regard to mouse studies: why the mAb and fentanyl were administered IP instead of a more relevant route of administration such as SC or IV? But I then acknowledge that Figure S3 has data obtained with IV challenges of fentanyl and antibody in rescue.

Only in the antinociception studies (Fig 2 and S2) was IP fentanyl used because it produces a reliable and consistent level of response that has been optimized by our group. Also, the antinociception prevention experiment (Fig S2) contained IP mAb dosing as the initial screening assay for mAb selection. Because antinociception is alredy not the most translationally relevant assay, we felt the IP route was acceptable for the initial proof of concept studies, which were then followed up with IV dosing in mouse studies (Fig 2C, Fig S3) as well as highly translational NHP studies.

2) In regards to “Blood samples for determination of anti-CSX-1004 antibodies (via anti drug antibody ELISA)”, and the related description of ADA detection on page 10. The coating antigen Fentanyl-BSA + CSX-1004 as detection setup would be very effective in detection of anti-CSX-1004 antibodies directed against the Fc portion of the CSX-1004, but less so for detection of Fv portion of the CSX-1004. Please discuss this possibility of underestimating the detection of ADA.

This is a good point and it is certainly possible that the assay is biased toward detecting anti-Fc ADAs. However, already the assay is of limited translational value because of the species incompatibility between a non-human rat model and the human mAb; thus, ADA formation toward a human mAb is more probable in a rat vs. humans. We have mentioned this limitation now in the main text. For our phase I clinical trial we have developed a different assay format which is not biased toward the Fc region: a sandwich ELISA in which capture and detection reagents are based on CSX-1004 and the analyte is the ADA.

“However, the ADA results are limited because of the increased immunogenicity of a human mAb dosed in a rat, and the specific assay format may be biased toward detecting ADAs only against the antibody Fc portion. Thus, further assay development work must be performed for clinical testing.”

3) Table S4. Did the studies yield sufficient timepoints to measure ED50 values ± SD and the shift pre- post- administration of the antibody?

Yes, however, ED_{50} calculations were not reliable for the NHP timecourse experiment (Fig 4) because at many of the timepoints a near complete blockade was observed at all fentanyl doses tested; thus, ED_{50}s could not be determined because a full dose response curve was not observed. For experiments in which we obtained full dose response curves pre- and post-mAb as well as post-washout (Fig 5 and 6), ED_{50}s ± SDs are provided in Tables S4 and S5.

4) Table S5: pre-mAb vs post- ED50 ± SEM (mg/kg) shows that mAb doesn’t bind morphine, oxycodone, and alfentanil. Is there an explanation that alfentanil was not bound?

Yes, as shown in Fig 1, CSX-1004 binding to alfentanil was virtually undetectable which correlates to the results we observed in vivo (Fig 6). We have clarified this in the text. Essentially it is due to the key structural change in fentanyl/carfentanil vs. alfentanil in which the latter contains an atypical change in the molecule’s ‘tail’ consisting of a tetrazolone which abrogates mAb binding vs. the typical phenethyl ‘tail’ found in most fentanyl analogs. Such result is not unexpected since the immunizing hapten molecule also contains this typical phenethyl group (Fig 1), not the atypical tetrazolone.

“R_6 substitution with the methyl ester or a tetrazolone derivative found in remifentanil and alfentanil, respectively, almost entirely abrogated antibody binding, potentially due to the disruption of van der Waals interactions between the phenethyl “tail” and the hydrophobic binding pocket when replaced with more polar groups.[23, 25]”

"While alfentanil is considered a FA, the atypical tetrazolone-containing "tail" instead of the typical phenethyl "tail" appeared to nullify any interaction with CSX-1004 both in vitro and in vivo; however, neither alfentanil nor any other analogs with a derivatized "tail" have been detected in the illicit drug supply. In addition, the lack of CSX-1004 interaction with alfentanil preserves the use of alfentanil for anesthesia in CSX-1004 treated patients."

Reviewer #2 Summary:
This study investigated ability of a CSX-1004, a fentanyl targeting monoclonal antibody, to reverse and prevent clinically relevant effects of opioids, particularly antinociceptive and respiratory-depressant effects, as well as characterize pharmacokinetic and toxicological profile. Using several relevant procedures in different species, including nonhuman primates (squirrel monkeys), these studies showed that CSX-1004 selectively blocked effects of fentanyl and carfentanil, without altering effects of non-fentanyl drugs such as oxycodone and morphine, and did so in time- and dose-related manner. Overall, the set of studies are rigorously conducted using relevant procedures with cross-species and within-subject generalization of results. Different behavioral endpoints and dose-response functions were employed to more fully characterize interactions between the antibody and opioids. Importantly, studies demonstrate effectiveness in primates increasing confidence in successful translation of these results to the clinic. This paper will make an important contribution to the literature and should be of interest to a broad readership. Below are a few comments for consideration in revision.

1) "…to rescue mice from fentanyl-induced antinociception and respiratory depression." Reconsider the use of rescue here, especially as it relates to antinociceptive effects. Perhaps "reverse"?

We agree and have changed the word to "reverse".

2) "A rapid reversal…within 20 minutes…" Not sure this should be characterized as "rapid", given the need for quick reversal of fentanyl effects noted in the Introduction.

We agree and have removed the word "rapid" and changed it to "marked".

3) "While naloxone was initially more effective at reversing respiratory-depression, CSX-1004 markedly surpassed the magnitude of naloxone…" Might not a larger dose of naloxone be expected to produce a greater magnitude of effect (i.e., full reversal)? The comparison of magnitude of effect in the absence of dose-effect relationships, including an asymptote at the maximum effect, is tenuous.

The reviewer makes a good point that a larger doses of naloxone would likely produce a greater effect; however, the same is true for the mAb as we have demonstrated in NHPs. Furthermore, the dose we used in the mouse study (1 mg/kg) is a very high dose of naloxone compared to what is given as an injection to humans (2 mg ≈ 0.025 mg/kg). We will clarify this point in the text.

"While higher doses of naloxone above the 1 mg/kg dose used in our study may increase the magnitude of OIRD reversal, the same would be expected of CSX-1004 according to our NHP

studies demonstrating dose dependent efficacy. However, the standard naloxone dose by injection in humans of approximately 0.025 mg/kg is 40 times less than the dose we used in mice; thus, the benefit of higher naloxone in our particular mouse model may be limited."

4) Figures 2a and 2b: recommend having ticks on the actual time points, rather than forcing the reader to interpolate.

Thank you for the suggestion. Unfortunately not all tick marks fall on an even multiple of one number, but we have changed the number interval on the x-axis to 15 in Figs 2a/2b as is consistent with 2c.

5) Results note differences in the magnitude of antagonism. Were shifts compared statistically against each other, or are these conclusions based on nominal differences in the mean for the group?

The statistical significance in fold-shifts was calculated by comparison of group mean opioid ED_{50} ± SDs at baseline, after mAb treatment and following a washout period (if applicable) by a one-way ANOVA with Dunnett's post-hoc test to compare either 10 or 40 mg/kg mAb vs. baseline. We have noted this in the methods section.

"…as group means ± SD before and after mAb treatment…"

REVIEWERS' COMMENTS

Reviewer #1 (Remarks to the Author):

All comments/concerned were addressed

Reviewer #2 (Remarks to the Author):

Revisions have addressed all of my earlier comments adequately.

There is a place in revised text where I believe a word is missing: "...thus, the benefit of higher naloxone in our particular mouse model may be limited."

Other than that, I have no further concerns.

Nice work. Thanks.

Revision #2

<u>Response To Reviewers:</u>

<u>Reviewer #1:</u>

1) All comments/concerned were addressed

We thank the reviewer for his or her time and input.

<u>Reviewer #2</u>

1) Revisions have addressed all of my earlier comments adequately.

There is a place in revised text where I believe a word is missing: "...thus, the benefit of higher naloxone in our particular mouse model may be limited."

Other than that, I have no further concerns.

Nice work. Thanks.

We thank the reviewer for his or her time and input. We have inserted the missing word in the revised manuscript.

"…thus, the benefit of higher naloxone doses in our particular mouse model may be limited."
